# Slim cigarette smoking in Urban China: Who are the early adopters and why?

**DOI:** 10.1371/journal.pone.0254682

**Published:** 2021-07-13

**Authors:** Jijiang Wang, Shiushing Wong, Yue-Lin Zhuang, Yuan Jiang, Shu-Hong Zhu

**Affiliations:** 1 Moores Cancer Center, University of California, San Diego, La Jolla, California, United States of America; 2 Tobacco Control Office, Chinese Center for Disease Control and Prevention, Beijing, China; Medical University of South Carolina, UNITED STATES

## Abstract

Sales data in China indicate that slim cigarette consumption has increased dramatically over the last few years. This study examined who smoked slim cigarettes and the reasons for adopting these new products. A survey of an online panel from 19 Chinese cities was conducted from October 2018 to April 2019 with 20,055 members aged 16 and older. Among the 31.7% [95% confidence interval (CI) = 30.1–33.4] of panel members who reported currently smoking, 37.7% (95% CI = 34.8–40.5) smoked slim cigarettes. Among smokers, women were significantly more likely to smoke slim cigarettes than men [56.5% (95% CI = 50.8–62.2) vs. 35.5% (95% CI = 32.8–38.1)]. Smokers with a bachelor’s degree were more likely to smoke slim cigarettes than those without [41.3% (95% CI = 38.1–44.4) vs. 33.1% (95% CI = 30.0–36.1)]. Most slim cigarette smokers were dual smokers [77.7% (95% CI = 75.3–80.1)], smoking both regular and slim cigarettes. Among dual smokers, 97.5% (95% CI = 96.7–98.3) started smoking regular cigarettes before slim cigarettes. Of the many reasons given for smoking slim cigarettes, 37.0% (95% CI = 34.3–39.7) directly related to harm reduction with another 10.1% (95% CI = 8.4–11.7) reporting their reason as wanting “to reduce consumption of regular cigarettes,” a plausible indication of harm reduction. These findings suggest strong interest in harm reduction among the current Chinese smoking population and that the popularity of slim cigarettes is likely to increase, with the more educated as the early adopters. Given the absence of any evidence that these products actually reduce harm, it is urgent that the public health community be on high alert in order to avoid repeating the sad history of low-tar cigarettes, when a supposed harm-reduction product misled the field of tobacco control.

## Introduction

Slim cigarettes have a smaller circumference than regular cigarettes. Although other versions preceded it, slim cigarettes became popular following the successful release of Virginia Slims, a brand first manufactured by Philip Morris in the U.S. in 1968 [1]. Subsequent decades have witnessed further development by tobacco companies of slim cigarettes with different circumferences that are all smaller than regular cigarettes (demislims, superslims, and microslims) [2]. Criteria for slim cigarettes vary by country. For example, the Chinese tobacco industry defines cigarettes with a circumference of 17mm ± 1mm as slim cigarettes [3], which fall into the category of superslim cigarettes in some other countries. While various nations have regulations restricting the packaging and appearance of cigarettes, fewer of them have set standards for the circumference [4–6]. Earlier studies have found that the introduction and promotion of slim cigarettes in several English-speaking countries were associated with the increase of women there smoking [7–11]. More recent studies, including surveys, focus groups, and analysis of industry strategies, have found that the interest in slim cigarettes is not limited to English-speaking countries [8, 12–16]. For example, 2017 sales data showed substantial market shares of slim cigarettes in Russia (15.1%), Latvia (21.2%), Poland (27.9%), Belarus (30.5%), and South Korea (36.6%) [17]. Most of these countries have a high smoking prevalence, especially among men [18]. As such, for these countries it is likely that the target for slim cigarettes is no longer women only.

Slim cigarettes were introduced into the Chinese market in early 2000 [17]. At that time, industry documents issued by the Chinese State Tobacco Monopoly Administration (STMA) began to address the topic of harm reduction [19–21]. STMA is a centralized government agency that controls the manufacture and sale of tobacco products in China through executive orders and directives to its branches, provincial, city, and county levels of government. STMA also goes by the name China Tobacco National Corporation (CTNC) when acting in more of a corporate or sales capacity [22]. While these earlier STMA documents mentioned harm reduction, slim cigarettes were not yet specifically promoted as harm-reduction products [20, 21, 23]. Moreover, in 2011 there were only four Chinese slim cigarette brands, along with a few slim cigarette brands imported from other countries [17]. Then, STMA issued two documents in 2014 and 2015, specifically for slim cigarettes, to its provincial and county-level agencies and affiliated companies [3, 24]. In these documents, they were instructed to develop and manufacture slim cigarettes as “low harm, high quality” products, and promote the sales of slim cigarettes as such. Given that tobacco advertisements were prohibited in China, the main channel of tobacco product marketing is through the tobacco retail system, which is regulated by STMA. This allows the system to promote tobacco products with various messages such as “low tar” and “high quality” [25–27].

By 2018, the number of slim cigarette brands had increased to 42 [28]. In addition to brand variety, the market share for slim cigarettes in China increased significantly [17]. One study reported that the market share of slim cigarettes grew from only 0.1% in 2012 to above 5% in 2017 [29]. The sales data in the first quarter of 2019, however, indicated that slim cigarettes reached 10% of all cigarettes sold in China [30, 31].

Before the 2014–15 documents promoting slim cigarettes, CTNC had developed a tobacco Premiumization Strategy in 2009 [32–34]. That strategy aimed to replace low-priced brands with higher-priced premium brands. Recognizing that economic growth had increased the affordability of cigarettes for Chinese smokers, CTNC used the Premiumization Strategy to encourage smokers to trade up. Premium brands were promoted as higher quality, thus meriting a higher price. Moreover, the premium brands tended to have lower levels of tar than the existing lower-priced brands, suggesting a lower level of harm. The strategy was apparently very successful: the proportion of smokers who reported consuming premium brands increased from 8.8% in 2009 to 32.1% in 2013–2015 [33]. Interestingly, even though the main promotional message of the Premiumization Strategy was on the higher quality of these higher-priced cigarettes, smokers still associated “higher quality” with “lower harm” [33].

Since STMA issued the 2014–15 documents, slim cigarettes have essentially been promoted in China with two chief messages. First, slim cigarettes are deemed higher quality because most of them are premium brands [35]. Second, they are explicitly billed as harm-reduction products [19–21, 23]. The sales data suggest that slim cigarettes have benefited from both messages. While the market share for premium brands increased from 33.4% in 2015 to 40.4% in 2018, the proportion of slim cigarettes among all premium brands sold increased from 4.3% to 18.0% during the same period [28, 36–39]. It seems that smokers in China have increasingly picked up the harm-reduction message even though there is no evidence that the harm of smoking slim cigarettes is lower than that of regular cigarettes. This is reminiscent of the history of low-tar cigarettes, and it ought to worry the public health community [40, 41]. However, the tobacco control community in China has so far made few official comments on slim cigarettes.

Smokers in China are predominantly male, with 50.5% of men and 2.1% of women currently smoking [42]. While the dramatic increases seen in the sales data suggest that slim cigarettes must have been consumed by more than just female smokers in China [29], few studies have examined the characteristics of slim cigarette smokers. If men are smoking slim cigarettes, what are their reasons for using them? If STMA views slim cigarettes as potentially harm-reducing products, do smokers perceive them as less harmful than regular cigarettes?

This study examined slim cigarette use among Chinese urban populations. It described the demographic characteristics of those who smoke slim cigarettes and examined the consumption pattern of slim cigarette smokers, including whether they started smoking regular cigarettes before slim cigarettes. The study also examined the risk perception of slim cigarettes and explored the motivation behind their use. It aimed to provide a first analysis of the use of these relatively new tobacco products in China, where more than 300 million people currently smoke [42].

## Methods

### Sample and setting

The online survey for this study was based on an established panel by ePanel®, one of the leading marketing research companies in China (http://group.epanel.cn/en/index.html) [43]. The panel contains about 1.8 million members, including long-term residents and short-term migrants, from major cities in China. Those members were recruited through advertisements via the internet, traditional media, and social media [44]. Thus, these panel members are a convenience sample of city residents, not a probability sample of the Chinese urban population. They tend to be younger and have higher educational attainment than the general urban population, as has been shown by social science research and commercial marketing research that have used the panel [44].

The present study recruited participants among panel members in 19 major Chinese cities, all of which had at least 10,000 members represented in the panel. Geographic location was taken into account in order to balance regional distribution. The cities selected were Beijing, Shanghai, Guangzhou, Shenzhen, Hangzhou, Chengdu, Chongqing, Dalian, Ha’erbin, Qingdao, Shenyang, Tianjin, Xi’an, Zhengzhou, Fuzhou, Nanchang, Wuhan, Nanning, and Changsha. Participants had to be at least 16 years old. Altogether, there were 552,652 qualified panel members from these 19 cities. A total of 359,698 were randomly sampled from the qualified members, with about half of them from the four most developed (Beijing, Shanghai, Guangzhou, and Shenzhen).

The survey was conducted from October 2018 to April 2019. Those sampled were sent invitations to participate, with the survey link provided via email and/or text message. Overall, 20,506 (5.7%) completed the survey. The analysis excluded 451(2.2%) respondents for failing one of the two questions embedded for attention checking during the survey process. This resulted in a final effective sample of 20,055. Informed consent was acquired from all participants, who agreed to participate in the online survey voluntarily. The online survey was approved by the UCSD Human Research Protections Program and Institutional Review Board of Chinese Center for Disease Control and Prevention.

The participants in the survey were younger than the general urban population of China [45], with 80.9% of them being younger than 40 years old. Specifically, the proportion of participants who were aged under 25, 25–29, 30–34, 35–39, and 40 and above were 18.4%, 26.3%, 21.1%, 15.1%, and 19.1%, respectively. Among the participants, 59.0% were male. Additionally, the participants were relatively educated, with 57.8% of them having earned a bachelor’s degree or higher.

### Measures

Current smokers were defined as people who had smoked at least 100 cigarettes in their lifetime and currently smoked cigarettes every day or on some days. The type(s) of cigarettes smoked was assessed by asking the question “What kind of cigarettes do you currently smoke?” with response options of “regular cigarettes,” “slim cigarettes,” and “both.” Accordingly, smokers were coded as exclusive regular cigarette users, exclusive slim cigarette users, and dual smokers. Smokers who had ever smoked both slim cigarettes and regular cigarettes were asked which type of cigarettes they smoked first.

Intensity of cigarette use was measured by the number of days per month smokers used regular and/or slim cigarettes, and the number of regular and slim cigarettes consumed per day on the days they smoked. The total number of cigarettes used per day by dual smokers was obtained by adding the number for regular cigarettes with that for slim cigarettes.

Current slim cigarette users were asked about their chief reason for using slim cigarettes, with answer options of “less harmful than regular cigarettes,” “reduce the consumption of regular cigarettes,” “help quit regular cigarettes,” “look good,” “taste good,” “cheaper than regular cigarettes,” and “other reasons.” For each respondent, only one reason could be selected from the list. As few selected “cheaper than regular cigarettes” as the reason, this category was collapsed with “other” in the analysis. All participants were asked about the risk of using slim cigarettes and regular cigarettes on a 10-point Likert scale ranging from “completely harmless” (1) to “extremely harmful” (10).

### Statistical analyses

Rates and proportions were calculated for measures for all participants and subgroups. The 95% confidence intervals were also calculated and presented along with rates and proportions [46]. Since the study participants were recruited from convenience samples from various cities, and this study focused on examining the data pattern within the recruited sample rather than attempting to estimate any population prevalence, no attempt was made to weigh the results by city. However, the analysis did include consideration of clustering effects by city in computing the confidence intervals for the point estimates. Finally, a multiple logistic regression was conducted to test the predictive values of risk perception, controlling for the effects of demographics. All analyses were conducted using SAS 9.4 (Research Triangle Institute, Research Triangle Park, NC).

## Results

Table 1 shows that 31.7% of the survey participants were current smokers, with 19.8% exclusively smoking regular cigarettes, 2.7% exclusively smoking slim cigarettes, and 9.3% smoking both slim cigarettes and regular cigarettes (dual smokers). In other words, more than three quarters of the slim cigarette smokers were dual smokers (9.3/(2.7+9.3) > 75%). Among all smokers, 37.7% were slim cigarette smokers (the last column of Table 1).

**Table 1:** Percentages of current regular cigarette and slim cigarette smokers and proportion of slim cigarette smokers among all smokers.

	sample size	All cigarette smokers	Exclusive regular cigarette smokers	Exclusive slim cigarette smokers	Dual smokers (regular and slim cigarettes)	Proportion of slim cigarette users among all cigarette users
		*(a)*	*(b)*	*(c)*	*(d)*	*(e)*
		P (95%CI)	P (95%CI)	P (95%CI)	P (95%CI)	P (95%CI)
**All respondents**	20055	31.7 (30.1–33.4)	19.8 (18.3–21.2)	2.7 (2.2–3.1)	9.3 (8.5–10.1)	37.7 (34.8–40.5)
**Sex**						
Male	11868	48.0 (44.6–51.3)	31.0 (28.6–33.3)	3.3 (2.7–3.9)	13.7 (12.3–15.1)	35.5 (32.8–38.1)
Female	8187	8.2 (7.3–9.1)	3.6 (3.1–4.0)	1.7 (1.4–2.1)	2.9 (2.3–3.5)	56.5 (50.8–62.2)
**Age**						
<25	3681	21.5 (19.9–23.0)	12.0 (10.3–13.7)	2.0 (1.6–2.5)	7.4 (6.1–8.8)	44.1 (37.4–50.8)
25–29	5274	31.1 (29.1–33.1)	19.1 (17.3–21.0)	2.7 (2.2–3.2)	9.3 (8.1–10.5)	38.5 (34.7–42.4)
30–34	4240	37.5 (35.5–39.4)	23.3 (22.0–24.6)	3.5 (2.6–4.4)	10.7 (9.7–11.6)	37.8 (35.5–40.2)
35–39	3034	40.5 (37.0–43.9)	26.3 (23.3–29.3)	2.6 (1.9–3.2)	11.6 (10.2–13.0)	35.1 (31.6–38.6)
≥40	3826	29.2 (25.6–32.7)	19.1 (16.2–22.0)	2.4 (1.7–3.1)	7.7 (6.4–8.9)	34.4 (29.9–38.9)
**Education**						
No bachelor’s degree	8453	33.0 (30.2–35.7)	22.1 (20.0–24.1)	2.5 (1.9–3.1)	8.4 (7.3–9.5)	33.1 (30.0–36.1)
Bachelor’s degree or higher	11600	30.8 (29.5–32.2)	18.1 (16.8–19.4)	2.8 (2.3–3.2)	9.9 (9.0–10.9)	41.3 (38.1–44.4)

Note: a = b+c+d; e = (c+d)/a. The proportion (e) is computed directly from the data and then rounded to one decimal point.

Table 1 also presents the cigarette use patterns by demographic characteristics. The overall smoking prevalence was much higher among males, 48.0%, than females, 8.2% (p<0.001). Similarly, the prevalence of smoking slim cigarettes was higher among males (17.0% = 3.3%+13.7%), than females, (4.6% = 1.7%+2.9%, p<0.001). Proportionally, however, slim cigarettes were much more commonly used among female smokers, 56.5%, relative to male smokers, 35.5% (p = 0.003).

The smoking prevalence generally increased with age and peaked at 40.5% among people aged 35 to 39, then dropped with the group aged 40 and above. The prevalence of using slim cigarettes ranged from 9.4% (2.0%+7.4%) for people younger than 25 years old to 14.2% (2.6%+11.6%) for the 35–39 age group. The proportion of slim cigarette use among current smokers was highest among those who were younger than 25 years old.

Smoking prevalence was lower among those who had earned a bachelor’s degree or higher compared to those who had not. However, the proportion of slim cigarette users was much higher among those college graduates (41.3% vs. 33.1%; p = 0.009).

Table 2 shows cigarette consumption by current smokers. Exclusive regular cigarette users smoked on more days in a month (of the last 30 days) than exclusive slim cigarette users, 17.8 vs. 12.5 days. However, the average number of cigarettes smoked per day (CPD) was similar (10.4 vs. 10.0). Dual smokers reported smoking regular cigarettes on 15.3 of the past 30 days and slim cigarettes on 8.1 out of 30 days. It is not clear how many of those days are overlapping. On the days that dual smokers smoked regular cigarettes, they consumed an average of 8.1 CPD, and on the days they smoked slim cigarettes they consumed 5.4 CPD.

**Table 2:** Level of cigarette consumption among current smokers.

	N	Days smoking cigarettes in the last 30 days	Number of cigarettes smoked per day
		Mean (95% CI)	Mean (95% CI)
**Exclusive regular cigarette users**	3966	17.8 (17.1–18.5)	10.4 (10.0–10.8)
**Exclusive slim cigarette users**	534	12.5 (11.4–13.5)	10.0 (9.1–10.9)
**Dual smokers**			
Regular cigarettes	1863	15.3 (14.2–16.4)	8.1 (7.4–8.8)
Slim cigarettes	1863	8.1 (7.3–8.9)	5.4 (5.0–5.9)

Using the data on the number of smoking days and cigarettes smoked per day presented in Table 2, we can estimate that the exclusive regular cigarette smokers smoked about 185 cigarettes per month, exclusive slim smokers about 125 cigarettes, and the dual smokers about 168 cigarettes per month.

A rough estimate of the proportion of slim cigarettes consumed among total cigarette consumption can be obtained by using the information presented in Tables 1 and 2. The total number of regular cigarettes consumed by exclusively regular cigarette smokers was 735,091 (20055×19.8%×10.4×17.8). The total number of slim cigarettes consumed by exclusive slim cigarette smokers was 67,686 (20055×2.7%×10.0×12.5). The total number of regular cigarettes consumed by dual smokers was 231,144 (20055×9.3%×8.1×15.3). The total number of slim cigarettes consumed by dual smokers was 81,580 (20055×9.3%×5.4×8.1). Thus, the monthly number of cigarettes consumed by all smokers was 1,115,501 (735,091+231,144+81,580+67,686), and the proportion of slim cigarettes among all cigarettes consumed was about 13.4% (81,580+67,686/1,115,501).

Table 3 shows the proportion of current slim cigarette smokers (exclusive and dual) who started smoking regular cigarettes first. Among those who currently smoked both regular and slim cigarettes, 97.5% started with regular cigarettes. Among those who currently only smoked slim cigarettes, 41.9% reported that they first smoked regular cigarettes. On average, therefore, about 85% of all slim cigarette smokers started their smoking habit with regular cigarettes.

**Table 3:** Proportion of current slim cigarette smokers who initiated with regular cigarettes.

	Overall		Male		Female	
	N	P(95% CI)	N	P(95% CI)	N	P(95% CI)
**All slim cigarette users**	2397	85.1 (83.3–86.9)	2018	87.0 (85.2–88.8)	379	74.9 (70.9–79.0)
**Dual smokers**	1863	97.5 (96.7–98.3)	1627	98.1 (97.4–98.8)	236	93.2 (91.0–95.4)
**Exclusive slim cigarette users**	534	41.9 (37.7–46.2)	391	40.9 (35.9–45.9)	143	44.8 (37.7–51.8)

The same initiation pattern is found for male and female slim cigarette smokers. For both men and women, the overwhelming majority of current dual smokers (98.1% and 93.2%) started with regular cigarettes. For those who currently only smoked slim cigarettes, 40.9% of men and 44.8% of women reported starting with regular cigarettes.

Table 4 shows the reported reasons for smoking slim cigarettes. The top three selected reasons were that slim cigarettes were “less harmful than regular cigarettes” (32.2%), they “look good” (28.5%), and they “taste good” (21.1%). Overall, 37.0% (32.2% + 4.8%, 95% CI = 34.3–39.7) of the reasons chosen could be considered directly related to harm reduction (“less harmful” for 32.2% and “help quit regular cigarettes” for 4.8%). Another 10.1% reported their reason as “reduce the consumption of regular cigarettes,” which could be attributed to a desire for harm reduction or for other reasons.

**Table 4:** Chief reason to smoke slim cigarettes.

Reasons for smoking slim cigarettes	Overall	Male	Female
	(N = 2397)	(N = 2018)	(N = 379)
	P (95%CI)	P (95%CI)	P (95%CI)
Less harmful than regular cigarettes	32.2 (29.5–35.0)	33.3 (30.9–35.7)	26.4 (19.4–33.4)
They look good	28.5 (26.2–30.9)	27.4 (25.0–29.7)	34.8 (29.1–40.5)
They taste good	21.1 (18.6–23.6)	20.8 (18.0–23.6)	22.7 (19.5–25.9)
Reduce the consumption of regular cigarettes	10.1 (8.4–11.7)	9.9 (7.9–11.8)	11.1 (8.5–13.7)
Help quit regular cigarettes	4.8 (3.6–6.0)	5.3 (4.0–6.6)	2.1 (0.0–4.5)
Other	3.3 (2.6–4.0)	3.4 (2.8–4.0)	2.9 (1.3–4.5)

There is a gender difference in reported reasons for smoking slim cigarettes. More men used slim cigarettes for harm reduction, 38.6% (33.3% + 5.3%), than women, 28.5% (26.4% + 2.1%, p <0.01). More than a third of women, 34.8%, opted for “look good” as the main reason for using slim cigarettes, which is significantly higher than the proportion among men, 27.4%.

**Fig 1:**
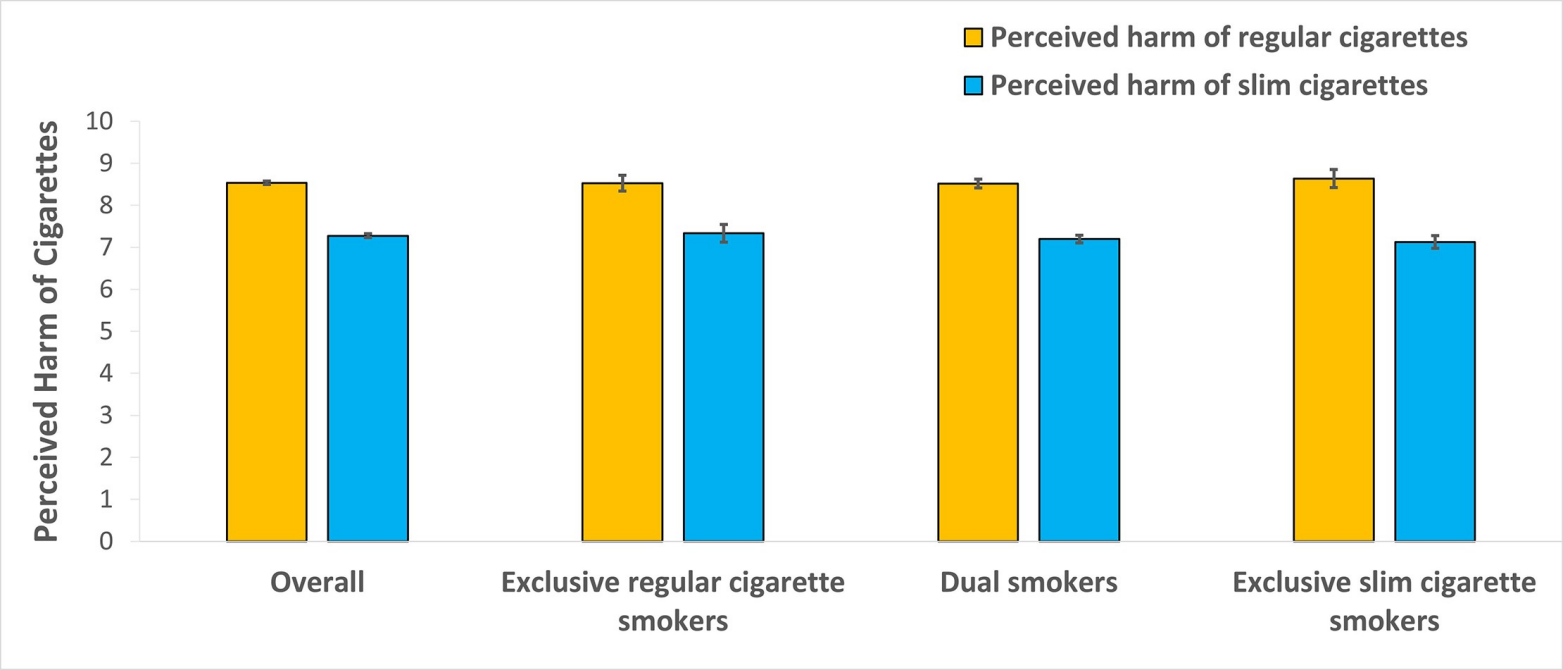
The perceived harm of regular and slim cigarettes among current smokers (10-point likert scale).

Fig 1 depicts the perceived harm of regular cigarettes and slim cigarettes. Overall, the perceived harm of slim cigarettes was significantly lower than that of regular cigarettes (7.3 vs 8.5 on a 10-point scale, p<0.001). When examined by subgroups (exclusive regular cigarette smokers, dual smokers, or exclusive slim cigarette smokers), all were found to perceive slim cigarettes as significantly less harmful than regular cigarettes: the risk ratings for the two products were 7.3 vs. 8.5, 7.2 vs. 8.5, and 7.1 vs. 8.6, for exclusively regular cigarette smokers, dual smokers, and slim cigarette smokers, respectively (all p values <0.001).

Finally, individual smokers were grouped by their perception of the relative risk of the two types of cigarettes: their rating of the harm of slim cigarettes and that of regular cigarettes could be the same, or one was greater than the other. Most rated slim cigarettes as less harmful than regular cigarettes (64%), with 31% rating them equally harmful, and 5% considering slim cigarettes to be more harmful. Then, their perception of relative risk was entered into a multiple logistic regression model to predict the likelihood of smoking slim cigarettes, controlling for the effects of demographics. The results are presented in Table 5.

**Table 5:** Factors associated with slim cigarette use among current smokers.

Variable	N	Adjusted OR (95%CI)	P
**Perceived harm of slim cigarettes relative to regular cigarettes**			<0.0001
Less harmful	12825	1.4 (1.3–1.6)	
Equally harmful	6261	Ref	
More harmful	969	1.1 (0.8–1.4)	
**Sex**			<0.0001
Male	11868	Ref	
Female	8187	2.3 (1.8–2.8)	
**Age**			<0.0001
<25	3681	1.5 (1.2–1.9)	
25–29	5274	1.0 (0.9–1.3)	
30–34	4240	1.0 (0.8–1.2)	
35–39	3034	1.0 (0.8–1.2)	
≥40	3826	Ref	
**Education**			<0.0001
No bachelor’s degree	8453	Ref	
Bachelor’s degree or higher	11600	1.4 (1.2–1.6)	

A multivariate logistic regression analysis (N = 20055).

Table 5 shows that those who rated slim cigarettes as less harmful than regular cigarettes were significantly more likely to be current smokers of slim cigarettes, compared to those who rated them equally harmful (Odds ratio = 1.4). If they rated slim cigarettes as more harmful, then there was no statistical difference in likelihood of smoking slim cigarettes. The multivariate logistic regression model also confirms the predictive values of demographic factors as shown in Table 1. That is, among smokers regarding the use of slim cigarettes: females were significantly more likely than males to smoke slim cigarettes; those under 25 were more likely than older ages, and those who had a college degree were more likely to smoke slim cigarettes than those who had no college degree.

## Discussion

The present study found that more than a third of Chinese urban smokers are currently smoking slim cigarettes. This is a much larger segment of the smoking population than the cigarette sales data published by the CNTC might suggest. For instance, sales data in the first quarter of 2019 indicated that slim cigarettes reached 10% of all cigarettes sold in China [30, 31]. Based on samples from 19 Chinese cities, this study found that about 38% of current smokers smoked slim cigarettes. This apparent discrepancy, as will be discussed in detail below, suggests that many slim cigarette smokers are early adopters who are still experimenting with these new products and that it is likely consumption on the individual and population level will increase.

A notable finding is that most slim cigarette users were dual smokers (77.7%), and most of those dual smokers used regular cigarettes before slim cigarettes (97.5%). There can be multiple reasons why regular cigarette smokers chose to also smoke slim cigarettes after having taken up regular cigarettes. One reason is that slim cigarettes were newer products. Most current dual smokers were already smokers when slim cigarettes came on the market. Novelty can be appealing and might have enticed regular smokers to expand their pattern of consumption. The fact that a substantial proportion of slim cigarette smokers selected “look good” as their chief reason to smoke slim cigarettes supports this interpretation.

However, the most significant motivation for smokers to choose slim cigarettes is the desire to reduce the harm attributed to smoking regular cigarettes. Most smokers perceived slim cigarettes as less harmful than regular ones (7.3 vs 8.5, Fig 1). The fact that they rated the harm of slim cigarettes at 7.3 on a 1 to 10 scale suggests that they still considered smoking slim cigarettes a significant health risk. However, they apparently believed switching would significantly reduce the risk. Nearly 40% of the slim cigarette smokers indicated that their main reason to smoke slim cigarettes was because they were less harmful. Therefore, for these smokers who choose slim cigarettes as a harm-reduction product, their dual use may indicate a transitional phase in which they experiment with slim cigarettes before eventually switching to them exclusively. The fact that more than 40% of exclusive slim cigarette smokers used to be regular cigarette smokers (Table 3) supports this possibility.

Another indication that dual smokers might be experimenting with slim cigarettes is that they tended to smoke slim cigarettes on fewer days, and smoke fewer slim cigarettes on the days that they did smoke, compared to exclusive slim cigarette smokers. Given that the overwhelming majority of the dual smokers started smoking regular cigarettes first (i.e., the dual smokers initially smoked zero slim cigarettes), it is plausible that they started by substituting some regular cigarettes with slim cigarettes on certain days and then gradually increased the number of days they smoked slim cigarettes and the number of slim cigarettes per day. At the same time, they might have decreased the consumption of regular cigarettes on the days that they smoked. Eventually, some dual smokers may switch completely to slim cigarettes, as indicated by the data in Table 3.

The current lower level of consumption of slim cigarettes among dual smokers also explains why the proportion of sales of slim cigarettes among total cigarette consumption (around 10% based on national sales data) tends to be much smaller than the proportion of slim cigarette smokers among the total number of smokers (about 38% among urban young adults according to this study). If these dual smokers continue to reduce their regular cigarette consumption by increasing consumption of slim cigarettes, then the total sale of slim cigarettes will continue to increase even if the proportion of slim cigarette smokers among total smokers does not.

Fig 1, however, suggests that the proportion of current smokers among all smokers is likely to increase. The majority of those who currently smoked only regular cigarettes believed that slim cigarettes were significantly less harmful than regular cigarettes, almost as much as the dual smokers did. In other words, if the promotional message for slim cigarettes presented by STMA and the lack of any counter efforts from the public health community continue, then the risk perception for slim cigarettes will continue to shift towards more trust in its harm-reduction possibility. The increasing proportion of slim cigarettes among premium brands in the sales data also suggest this general trend [28, 36–39].

Another notable finding is that slim cigarettes are more popular among the more educated smokers than among the relatively less educated smokers. It is true that the overall smoking prevalence was lower among those who were more educated. However, given someone is a smoker, the likelihood of smoking slim cigarettes was significantly greater among the higher educated than the lower educated. It is possible that the higher educated group was more inclined to take the harm-reduction approach if they perceived slim cigarettes to be less harmful [47–49].

Currently, there is no research literature supporting the belief that smoking slim cigarettes is less harmful than smoking regular cigarettes. However, this study shows that many smokers do believe slim cigarettes are less harmful, as suggested in some experimental studies [50, 51]. This belief could be based in part on the noticeable feature that slim cigarettes are much smaller in volume than regular cigarettes. All things being equal, consuming less tobacco per cigarette would be less harmful because the harm of using tobacco is dose-responsive [52]. However, no research has shown that the actual intake of harmful chemicals is lower from smoking slim cigarettes than from smoking regular cigarettes. The history of low-tar cigarettes in the U.S. provides ample warning for the danger of equating lower intake of harmful substances with apparent design features of the products [41].

It is possible that slim cigarette use in China may turn out to be like the low-tar cigarette case in the U.S. In 1964, the U.S. released its first Surgeon General Report declaring the hazards of smoking to the public [53]. In response, the tobacco industry developed low-tar cigarettes [54]. Low-tar cigarettes were promoted as “less harmful,” even though the data suggested that smokers would compensate in their actual smoking behavior (e.g., inhaling deeper) in order to maintain a consistent nicotine dosage [40, 55, 56]. However, the concept of harm reduction through lowering the tar yield was quickly and widely accepted by the public [40, 47, 57]. Many smokers, especially the more educated ones, switched to low-tar cigarettes [47]. The market share of low-tar cigarettes increased dramatically, from 2% in 1967 to 55.4% in 1987 and to 87.9% in 2016 [58]. Only decades later did the research demonstrate that smoking low-tar cigarettes was no less risky than smoking regular cigarettes [41]. By then, millions of smokers had been misled to believe that they were practicing harm reduction [40].

The present study does not provide direct evidence that slim cigarette use in China will produce outcomes resembling the low-tar scenario in the U.S. However, the similarity between the two cases (the rapidly increasing popularity of low-tar cigarettes in the U.S. and slim cigarettes in China) is so striking that it should seriously alarm the public health community. Presently, there is little research done about smoking slim cigarettes. Many questions are unanswered or not even asked. For example, is there any preliminary epidemiological evidence that smoking slim cigarettes is less harmful than smoking regular cigarettes? Is switching from regular cigarettes to slim cigarettes a preparation to quit smoking totally, or will it lead smokers to be content with the change so that the need to quit smoking becomes less urgent? Will the design of slim cigarettes become so attractive to women or to youth that many, who otherwise would not smoke, will start smoking slim cigarettes? These and many other questions need to be asked and addressed, urgently.

The study has several limitations. First, the survey was conducted not with a representative sample of the Chinese population, but with an online panel of 19 major cities in China. The respondents were relatively young and more educated urbanites, who are more likely to adopt new products. Thus, the proportion of slim cigarette use among smokers found in this study, 37.7%, may be an overestimate. However, a 2019 government report from the Chinese Center for Disease Control and Prevention (China CDC), based on a 2018 national tobacco survey, showed that 36.4% of smokers aged 15–24 and 39.2% of smokers aged 25–44 were smoking slim cigarettes [42]. These age ranges correspond to the majority of participants in our study, and their proportions were similar to what is found in the present study. The proportion of slim cigarette users among all smokers was 33.0% for the national survey, which was slightly lower than the present study (37.7%) [42]. Second, it was somewhat difficult for the dual smokers to recall which day of the month they smoked slim cigarettes, which day they smoked regular cigarettes, and which day they smoked both. Thus, the estimated proportion of slim cigarettes among all cigarettes consumed for these dual smokers might be inaccurate. The present study estimated that for these young and urban smokers, slim cigarettes accounted for 13.4% of their overall cigarette consumption. As noted previously, the most recently accessible data on cigarette consumption in China found that about 10% of cigarettes sold in the first quarter of 2019 were slim cigarettes. Given that participants of this study were younger than the general population, a little higher proportion of slim cigarette consumption based on this survey, 13.4%, than that based on the sales data, 10%, seems within the bounds of the expected difference. Third, the survey did not ask for the brands of cigarettes. As a result, it was not possible to examine how much smokers prefer slim cigarettes because they are slim in design and how much because they are premium brands. As mentioned in the introduction, smokers in China have increasingly switched to smoking premium brands (since implementation of the Premiumization Strategy in 2009) and slim cigarettes (since the promotion of slim cigarettes as harm reduction products with the 2014–15 STMA documents). Even though the sales data from 2015 to 2018 suggest that the market share of slim cigarettes increased at a higher rate than that for premium brands in general [28, 36–39]. an analysis of consumption behavior at the individual smokers’ level can help clarify the contribution of promotional messages of higher quality and lower harm. Finally, this is a cross-sectional study, which limited its ability to assess the transition from regular cigarettes to slim cigarettes and vice versa. The pattern of change from smoking regular cigarettes to slim cigarettes in this study was based on smokers’ recall. A longitudinal study that tracks the change in smoking patterns over time, including any change in smoking cessation, would provide a more reliable description of the impact of slim cigarettes on smoking behavior among the Chinese population.

What this study has provided, however, is a glimpse into a potentially dramatic scenario in which slim cigarette smoking becomes a dominant tobacco-use behavior in China. Whether such a change would be a public health gain or loss is unknown. Presently, there is surprisingly little research addressing this topic or even publicly expressed concern from the public health community in China. Studies on smokers’ perceptions of premium brands and low-tar cigarettes as products of reduced harm [33, 59] seem to have not generated sufficient concern among the public health community in China. The study on consumption data of slim cigarettes [29] and the 2019 China CDC summary data appear to be the first public reports on the topic of slim cigarettes, and there has been little research on the belief and behavior related to slim cigarette smoking or promotional activities thereof. In fact, it is difficult to find research reports from other countries where slim cigarettes have become popular (at least that is the case in terms of journal publications written in English) [60]. However, the findings of the present study with Chinese smokers are eerily reminiscent of the early phase of the low-tar cigarette history in the U.S., in which smokers and the public health community were led to trust an unproven harm-reduction product [40, 41]. It is hoped that this study will raise the urgency of this topic among the international community of public health researchers and practitioners.

## Supporting information

S1 Questionnaire (PDF)

S1 Data Data for Table 1. (CSV)

S2 Data Data for Table 2. (CSV)

S3 Data Data for Tables 3 and 4. (CSV)

S4 Data Data for Fig 1. (CSV)

S5 Data Data for Table 5. (CSV)

## References

[1] American Assn of Advertising Agencies, Leo Burnett Agency, Weinstein H. Papers from the 1969 A.A.A.A. Region Conventions: How an agency builds a brand—the Virginia Slims story. 28 Oct 1969 [cited 13 Jan 2020]. Available: https://www.industrydocuments.ucsf.edu/tobacco/docs/#id=gjnf0164

[2] McAdam K, Eldridge A, Fearon IM, Liu C, Manson A, Murphy J, et al. Influence of cigarette circumference on smoke chemistry, biological activity, and smoking behaviour. Regul Toxicol Pharmacol. 2016;82: 111–126. doi: 10.1016/j.yrtph.2016.09.010 27634061

[3] State Tobacco Monopoly Administration. Directive on regulating and supporting the development of slim cigarettes. China Tobacco Yearbook 2015. Beijing: China Economic Publishing House; 2015. p. 528.

[4] European Commission. Directive 2014/40/EU of the European Parliament and of the Council of 3 April 2014 on the approximation of the laws, regulations and administrative provisions of the member states concerning the manufacture, presentation and sale of tobacco and related products and repealing Directive 2001/37/EC. Off J Eur Union. 2014;L127: 1–38.

[5] Australian Government. Tobacco Plain Packaging Act 2011, An Act to discourage the use of tobacco products, and for related purposes, No. 148. Attorney-General’s Department; 2011 [cited 22 Feb 2021]. Available: http://www.legislation.gov.au/Details/C2011A00148

[6] Parliamentary Council Office. Smoke-free Environments Regulations 2017 (LI 2017/123)–New Zealand Legislation. 2017. Available: https://www.legislation.govt.nz/regulation/public/2017/0123/latest/whole.html

[7] Carpenter CM, Wayne GF, Connolly GN. Designing cigarettes for women: new findings from the tobacco industry documents. Addiction. 2005;100: 837–851. doi: 10.1111/j.1360-0443.2005.01072.x 15918814

[8] Toll BA, Ling PM. The Virginia Slims identity crisis: an inside look at tobacco industry marketing to women. Tob Control. 2005;14: 172–180. doi: 10.1136/tc.2004.008953 15923467 PMC1748044

[9] Holbert N. Cigarette tracking study: demographics of smokers. 1981. Available: https://www.industrydocuments.ucsf.edu/docs/nfdx0117

[10] Pierce JP, Lee L, Gilpin EA. Smoking initiation by adolescent girls, 1944 through 1988. An association with targeted advertising. JAMA. 1994;271: 608–611. 8301793

[11] US Department of Health and Human Services. Women and smoking: a report of the Surgeon General. Washington DC: 2001. Available: https://www.cdc.gov/tobacco/data_statistics/sgr/2001/index.htm

[12] Dewhirst T, Lee WB, Fong GT, Ling PM. Exporting an inherently harmful product: the marketing of Virginia Slims cigarettes in the United States, Japan, and Korea. J Bus Ethics. 2016;139: 161–181. doi: 10.1007/s10551-015-2648-7 28025588 PMC5181852

[13] Ford A, Moodie C, MacKintosh AM, Hastings G. Adolescent perceptions of cigarette appearance. Eur J Public Health. 2014;24: 464–468. doi: 10.1093/eurpub/ckt161 24158317

[14] Minaker LM, Tait H, Ong M, Nguyen N. Slim cigarette smoking prevalence among Canadian youth smokers: Implications for federal standardized packaging legislation. Can J Public Health. 2017;108: e565–e570. doi: 10.17269/CJPH.108.6197 29356665 PMC6972237

[15] Gallopel-Morvan K, Moodie C, Guignard R, Eker F, Béguinot E. Consumer Perceptions of Cigarette Design in France: A Comparison of Regular, Slim, Pink and Plain Cigarettes. Nicotine Tob Res. 2019;21: 911–917. doi: 10.1093/ntr/nty105 29800331

[16] Dewhirst T, Lee WB. Made For a Man Or a Woman? An Exploratory Comparison of Virginia Slims Advertising in the United States and Korea. ACR Asia-Pac Adv. 2005.

[17] Research Institute for Tobacco Economics. Analysis and thinking of the development of slim cigarettes. 2018 [cited 23 Aug 2019]. Available: http://www.tobacco.gov.cn/html/56/87679316_n.html

[18] World Health Organization. WHO report on the global tobacco epidemic 2019: Offer help to quit tobacco use. Geneva: 2019.

[19] State Tobacco Monopoly Administration.Guoyanke/2003/630 of the State Tobacco Monopoly Administration on Printing and Distributing the “China Cigarette Technology Development Outline.” 2003.

[20] State Tobacco Monopoly Administration. Guoyanke/2006/526 Outline of Medium and Long-term Development Plan for Tobacco Industry. 2006.

[21] State Tobacco Monopoly Administration. Guoyanke/2009/127 Opinions on Vigorously Promoting Cigarette Harm Reduction to Improve Technology Innovation. 2009.

[22] Overview of China Tobacco. [cited 22 Feb 2021]. Available: http://www.tobacco.gov.cn/gjyc/gkxx/202012/3525d1df961a4deda768db9db0034f70.shtml

[23] Xie B. Brief Analysis on the Slim Cigarette Market. 2017 [cited 27 Feb 2021]. Available: http://www.etmoc.com/market/Newslist?Id=37023

[24] State Tobacco Monopoly Administration. Directive on further accelerating the development of slim cigarettes. China Tobacco Yearbook 2016. Beijing: China Economic Publishing House; 2016. pp. 642–3.

[25] Li F. Anhui Provincial Tobacco Monopoly Administration promotes the cultivation of slim cigarette brands. 2015 [cited 13 Jan 2020]. Available: https://www.eastobacco.com/sypd/pppy/201506/t20150615_369525.html

[26] Zhuang J. How to effectively cultivate the slim cigarette market. 2019 [cited 13 Jan 2020]. Available: http://www.tobaccochina.cc/shangye/20195/20195219164_785939.shtml

[27] Marketing Department of Sanjiang County Tobacco Monopoly Administration. Marketing tips from talent retailers of new tobacco products. 2018 [cited 13 Jan 2020]. Available: http://www.tobacco.gov.cn/html/21/2105/210503/21050301/86870412_n.html

[28] Release of 2018 slim cigarette ecological report: What happened to the 2018 slim cigarette market? 2020 [cited 16 Jul 2020]. Available: http://www.cn-nong.com/news/hydt/1669.html

[29] Zhang X, Xu X, Xu M, Hu T. The impact of tobacco taxation policy on slim cigarette use and the growing popularity of slim cigarettes in China since 2014. Health (N Y). 2019;11: 711–20. doi: 10.4236/health.2019.116059

[30] Slim cigarette wholesale sales data, 2019 Jan—Apr. 22 Aug 2019 [cited 13 Jan 2020]. Available: http://www.yanb2b.com/news/jiage/3898.html

[31] National Bureau of Statistics of China. National data: Cigarette sales. [cited 13 Jan 2020]. Available: http://data.stats.gov.cn/easyquery.htm?cn=B01&zb=A030109&sj=2019C

[32] Yang Q. Talking about achieving a higher level of cigarette structure and profit and tax. 2009. Available: http://www.etmoc.com/look/Looklist?Id=18179

[33] Xu SS, Gravely S, Meng G, Elton-Marshall T, O’Connor RJ, Quah ACK, et al. Impact of China National Tobacco Company’s ‘Premiumization’ Strategy: longitudinal findings from the ITC China Surveys (2006–2015). Tob Control. 2019;28: s68–s76. doi: 10.1136/tobaccocontrol-2017-054193 30158207 PMC6445774

[34] State Tobacco Monopoly Administration. The 2010 National Tobacco Work Conference was Held in Beijing to Deploy Activities in 2010. 2010 [cited 26 Feb 2021]. Available: http://www.gov.cn/gzdt/2010-01/19/content_1514777.htm

[35] Henggang Science & Technology. Year-end Inventory: Top Ten Keywords in the Cigarette Market in 2018. 2019 [cited 26 Feb 2021]. Available: http://www.hgtobacco.com/front/view-a72d23390a7c44dda80ce506904a22be-05c6530e56f948dbace745f37671ffcc.html

[36] Xinhua Finance. Annual Report——Analysis of the National Cigarette Market in 2020 and Prospective for 2021. In: China Financial Information [Internet]. 2020 [cited 26 Feb 2021]. Available: http://news.xinhua08.com/a/20201230/1970246.shtml

[37] The Task for Meeting the Tobacco Sales Target is Arduous. Is the Winter for Tobacco Industry Coming? 2018 [cited 26 Feb 2021]. Available: http://market.chinabaogao.com/yanjiu/11213R1222018.html

[38] Yanhuasanyue. New Sales Ranking, Embracing the Era Dominated by Tier 1 and Tier 2 Cigarettes. 2019 [cited 27 Feb 2021]. Available: http://domo.tobaccochina.com/html/zxsd/syyy/546717.shtml

[39] Tan Q. Tobacco Consumables Have a Strong Recovery Trend, and Novel Tobacco Products Lead Cigarette Consumption New Fashion——In-depth Tobacco Industry Report on Tobacco Consumables and Novel Tobacco Products. 2018. Available: https://pdf.dfcfw.com/pdf/H3_AP201809261201941232_1.pdf?1537946726000.pdf

[40] Warner KE, Slade J. Low tar, high toll. Am J Public Health. 1992;82: 17–18. doi: 10.2105/ajph.82.1.17 1536326 PMC1694398

[41] Burns DM, Major JM, Shanks TG, Thun MJ, Samet JM. Smoking lower yield cigarettes and disease risks. Risks associated with smoking cigarettes with low machine-measured yields of tar and nicotine Smoking and tobacco control monograph no 13. Bethesda, MD: National Cancer Institute; 2001. pp. 65–158.

[42] Chinese Center for Disease Control and Prevention. National Adult Tobacco Survey executive summary 2018. Beijing: Chinese Center for Disease Control and Prevention; 2019.

[43] ePanel Inc. [cited 10 Jun 2020]. Available: https://www.epanel.cn/research/en/index.html

[44] Zhan D, Kwan M-P, Zhang W, Fan J, Yu J, Dang Y. Assessment and determinants of satisfaction with urban livability in China. Cities. 2018;79: 92–101. doi: 10.1016/j.cities.2018.02.025

[45] National Bureau of Statistics of China. China Statistical Yearbook 2019. Beijing: China Statistics Press; 2019.

[46] Fleiss J, Levin B, Paik M. Statistical inference for a single proportion. Statistical Methods for Rates and Proportions. John Wiley & Sons, Ltd; 2004. pp. 17–49. doi: 10.1002/0471445428.ch2

[47] Giovino GA, Tomar S, Reddy MN, Peddicord JP, Zhu B-P, Escobedo LG, et al. Attitudes, knowledge, and beliefs about low-yield cigarettes among adolescents and adults. Smoking and Tobacco Control Monograph 7: The FTC Cigarette Test Method for Determining Tar, Nicotine, and Carbon Monoxide Yields of US Cigarettes, Report of the NCI Expert Committee. Bethesda, MD: National Cancer Institute; 1996. pp. 39–57.

[48] Gilpin EA, Emery S, White MM, Pierce JP. Does tobacco industry marketing of ‘light’ cigarettes give smokers a rationale for postponing quitting? Nicotine Tob Res. 2002;4: S147–S155. doi: 10.1080/1462220021000032870 12573176

[49] Stellman SD, Garfinkel L. Smoking habits and tar levels in a new American Cancer Society prospective study of 1.2 million men and women. J Natl Cancer Inst. 1986;76: 1057–1063. 3458944

[50] Islam F, Thrasher JF, Szklo A, Figueiredo VC, Perez C de A, White CM, et al. Cigarette flavors, package shape, and cigarette brand perceptions: an experiment among young Brazilian women. Rev Panam Salud Publica Pan Am J Public Health. 2018;42: e5. doi: 10.26633/RPSP.2018.5 31093036 PMC6385815

[51] White CM, Hammond D, Thrasher JF, Fong GT. The potential impact of plain packaging of cigarette products among Brazilian young women: an experimental study. BMC Public Health. 2012;12: 737. doi: 10.1186/1471-2458-12-737 22943135 PMC3575335

[52] Doll R, Peto R, Wheatley K, Gray R, Sutherland I. Mortality in relation to smoking: 40 years’ observations on male British doctors. BMJ. 1994;309: 901–11. doi: 10.1136/bmj.309.6959.901 7755693 PMC2541142

[53] US Public Health Service. Smoking and Health: Report of the Advisory Committee to the Surgeon General of the Public Health Service. US Department of Health, Education, and Welfare; 1964.

[54] Pollay RW, Dewhirst T. The dark side of marketing seemingly “Light” cigarettes: successful images and failed fact. Tob Control. 2002;11 Suppl 1: I18–31. doi: 10.1136/tc.11.suppl_1.i18 11893811 PMC1766068

[55] Hurt RD, Robertson CR. Prying Open the Door to the Tobacco Industry’s Secrets About Nicotine: The Minnesota Tobacco Trial. JAMA. 1998;280: 1173–1181. doi: 10.1001/jama.280.13.1173 9777818

[56] Leavell N-R. The low tar lie. Tob Control. 1999;8: 433–437. doi: 10.1136/tc.8.4.433 10629251 PMC1759759

[57] Russell MA. Low-tar medium-nicotine cigarettes: a new approach to safer smoking. Br Med J. 1976;1: 1430–1433. doi: 10.1136/bmj.1.6023.1430 953530 PMC1640397

[58] Federal Trade Commission. Federal Trade Commission Cigarette Report for 2017 and Federal Trade Commission Smokeless Tobacco Report for 2017. 2017 [cited 14 Jan 2020]. Available: https://www.ftc.gov/reports/federal-trade-commission-cigarette-report-2017-federal-trade-commission-smokeless-tobacco

[59] Elton-Marshall T, Fong GT, Zanna MP, Jiang Y, Hammond D, O’Connor RJ, et al. Beliefs about the relative harm of “light” and “low tar” cigarettes: findings from the International Tobacco Control (ITC) China Survey. Tob Control. 2010;19 Suppl 2: i54–i62. doi: 10.1136/tc.2008.029025 20935197 PMC2976003

[60] Pinkas J, Kaleta D, Zgliczyński WS, Lusawa A, Wrześniewska-Wal I, Wierzba W, et al. The prevalence of tobacco and e-cigarette use in Poland: a 2019 nationwide cross-sectional survey. Int J Environ Res Public Health. 2019;16: 4820. doi: 10.3390/ijerph16234820 31801221 PMC6926521

