# Supplementary material for: Slim cigarette smoking in Urban China: Who are the early adopters and why?
Source: PLoS One. 2021 Jul 13;16(7):e0254682. doi: 10.1371/journal.pone.0254682 (PMC8277019; doi:10.1371/journal.pone.0254682)
Supplement: S1 Questionnaire — (PDF) [file pone.0254682.s001.pdf]

Copyright ©UCSD

China Survey Questionnaire
8/22/2018

For questions, please Contact:
Dr. Zhu (Szhu@health.ucsd.edu)
Center for Research and Intervention in Tobacco Control
UC San Diego

Page 1

我们希望了解您对香烟和电子烟的看法和使用习惯。无论您是否吸烟都请您参与调查。只要完成问卷就能得到同样的积分。您回答的内容和题数不会影响积分。最重要的是把您真实的情况告诉我们。大概需要 5-10 分钟。非常感谢!

We want to understand your opinion regarding, and the use of cigarettes and e-cigarettes. Whether you have used these products or not, please participate. All who complete the survey will receive the same number of points. Your choice of answers and the number of questions you answered will not affect your points. The most important aspect is to tell us your real opinion and how you have used the products. It takes about 5-10 minutes to complete the survey. Many thanks!

a. 我同意参与本次调查 Yes, I will participate **Q1**
b. 我不同意 No, I am not going to take the survey

Page 2

請記得：您回答的内容和题数不影响积分。最重要的是把您真实的情况告诉我们。

Please remember: Your choice of answers and the number of questions you answered will not affect your points. The most important aspect is to tell us your real opinion and how you have used the products.

Page 3

Your input affects our knowledge of tobacco prevalence. Some questions will require you to put thought into your responses. You may not know the answer to other questions. Please select “I Don’t Know” instead of guessing.

您的回答将直接影响我们对烟草产品及其使用的了解。有些问题需要您经过仔细思考后再回答。如果您对所问的问题没有肯定的答案，请选择“不知道”或“不清楚”。

I will do my best to answer thoughtfully and accurately.
我会尽最大努力,仔细思考后如实回答问题。 **Q2**

a. Yes 我会
b. No 我恐怕做不到 ***[end of the survey]***

Thank you for your participation! 非常感谢您的参与!

To ensure the quality of the survey, we may contact a small percentage of respondents to follow up on responses.
为了保证调查质量，我们有可能在调查结束后联系小部分本次调查的参加者以便了解他们的意见。

“next” key to start survey

[Everyone]

Q1 你对“室内公共场所完全禁烟”的看法是。。。。？ **Q3**

Do you agree with the idea of completely smoke-free at indoor public places?

a. 完全赞同 completely agree
b. 比较赞同 mostly agree
c. 不赞同也不反对 neutral

d. 比较反对 mostly disagree
e. 完全反对 completely disagree

[Everyone]
在你居住的城市，以下公共场所是完全禁烟，部分禁烟，还是完全不禁烟?
In the city where you live, is smoking banned completely, partially, or not at all in the following public places?

	完全禁烟	部分禁烟	完全不禁烟	不知道
Q2 室内工作场所 (indoor worksites) **Q4**	☐	☐	☐	☐
Q3 餐厅 (restaurants) **Q5**	☐	☐	☐	☐

[Everyone]
Q4 你工作的地方是在室内还是室外? **Q6**
Are you working indoors or outdoors?
a. 室内 indoors
b. 室外 outdoors
c. 不工作/已退休 not working/ retired

[Ask if Q4=a]
Q5 你工作的地方禁烟吗? **Q7**
Is smoking completely banned in your workplace?
a．完全禁烟 completely
b。部分禁烟 partially
c。完全不禁烟 not at all
d. 不太清楚 not sure

[Ask if Q4=a]
Q6 过去 30 天里有人在你工作的地方吸烟吗? **Q8**
In the past 30 days, is anyone smoking in your workplace?
a．有 Yes
b．没有 No
c．不知道 DK

[Everyone]
Q7 你家里对在室内吸烟有什么限制吗? **Q9**
Are there any smoking restriction rules in your house?
a．所有房间都不能吸烟 smoking is not allowed in any room
b．部分房间不能吸烟 can only smoke in some rooms
c．没有限制，所有房间都可以吸烟 no restriction

[Everyone]
Q8 你吸过香烟吗? **Q10**
Have you ever smoked a cigarette?
a. 吸过 Yes
b. 没有 No

[Everyone]

Copyright ©UCSD

电子烟是一种模仿卷烟的电子产品，使用电池将**烟油**加热雾化后吸食，有些称为蒸汽电子烟。外形多样，如：

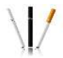 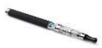 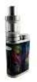

E-cigarettes are electronic devices that try to create a feeling of smoking cigarette. They work by heating e-juice to generate an aerosol that the user inhales . This type of e-cigarettes is also called “steam e-cig” in China. They come in many shapes, such as:

[Everyone]
Q9. 你用过这类使用**烟油**的电子烟吗？ **Q11**

Have you ever used E-cigarettes that use e-juice?

a. 用过 Yes
b. 没有 No

[Everyone]

Q10. 无烟烟草也称为口含烟，嚼烟，鼻烟 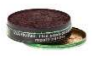，使用时将烟丝直接放在口腔内（如舌下，脸颊内侧）。你用过无烟烟草吗？ **Q12**

Smokeless tobacco can be made into snus, chew, or snuff. During use, shredded tobacco is placed directly in the mouth (eg, under the tongue, inside the cheeks). Have you ever used SLT?

a. 用过 Yes
b. 没有 No

[Ask if Q10=a]
Q10a 过去30天里你用过吗？ **Q13**

Have you used it in the past 30 days?

a. 用过 Yes
b. 没有 No

[Ask if Q10a=a]
Q10b 你用的是什么牌子？ **Q14**

Which brand did you use?

a. ______________ （请填入品牌） ***Please write down the brand name***
b. 不记得了 dk (**changed, 有改动**)

[Ask if Q10=a]
Q10c 你用过国产的长岛牌无烟烟草吗？ **Q15**

Have you ever used a Chinese brand called 长岛?

a. 用过 yes (**changed, 有改动**)
b. 没有 no
c. 不记得了 dk

[Everyone]

Q11. 尼古丁是香烟的主要成分之一，也是香烟能使人上瘾的原因。国外市场上有出售含有尼古丁的饮料 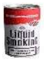 。你喝过这类尼古丁饮料吗？**Q16**

Nicotine is one of the main ingredients in cigarettes. It is also the reason people get addicted to cigarettes. Nicotine-containing drinks have been sold in foreign markets. Have you ever used nicotine drinks?

a. 喝过 Yes (**changed, 有改动**)
b. 没有 No

[Everyone]
Q12. 你喝过酒吗（如啤酒，红酒，白酒）？ **Q17**

Have you ever used alcohol (such as beer, red wine, white wine)?

a. 用过 Yes
b. 没有 No

[Everyone]
Q13 你的亲戚朋友当中现在有人吸烟吗？**Q18**

Among your relatives/friends, is anyone smoking?

a. 没有人吸烟 none of them
b. 少数人吸烟 few of them
c. 多数人吸烟 most of them
d. 全部都吸烟 all of them
e. 不清楚 dk

[Everyone]

香烟包括**普通卷烟**（如“中华”，“牡丹”，“红塔山”等） 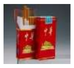

和**细支烟**（也称细烟，是比普通卷烟细的香烟。如“梦都（细支型）”，“南京（炫赫门）”等）。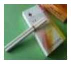
Cigarettes includes regular cigs, and slim ones …

[Everyone]
Q14 在参加这次调查以前你听说过细支烟吗？**Q19**

Before taking this survey, have you ever heard of slim cigarettes?

a. 听说过 yes
b. 没有 no

[Ask if Q14=a]
Q15 你的亲戚朋友当中现在有人吸细支烟吗？**Q20**

How many of your relatives/friends are using slim cigarettes?

a. 没有人吸 none of them
b 少数人吸 few of them
c. 多数人吸 most of them
d. 全部都吸 all of them
e. 不清楚 dk

[Ask if Q14=a]

Q16. 过去一个月里你的亲戚朋友当中有人谈论细支烟吗？**Q21**

In the past 30 days did anyone among your relatives/friends talk about slim cigarettes?

a. 有 yes

b. 没有 no

[Ask if Q16=a]

Q17 总的来说，他们对细支烟的态度是赞成还是反对？**Q22**

In general, what is their attitude toward slim cigarettes?

a．多数反对 mostly negative

b．多数赞成 mostly positive

c．中立 neutral

[Ask if Q14=a]

Q18 在过去一个月里你见过或听到过有关细支烟的新闻报道吗？**Q23**

In the past 30 days have you heard/read any news about slim cigarettes?

a. 有 yes

b. 没有 no

[Ask if Q18=a]

Q19 总的来说，这些新闻报道对细支烟的态度是赞成还是反对？**Q24**

In general what are their attitudes toward slim cigarettes?

a．多数反对 mostly negative

b．多数赞成 mostly positive

c．中立 neutral

[Ask if Q8=a]

Q20 前面您提到吸过香烟，您吸过哪一种烟？ What kinds of cigarettes have you ever smoked? **Q25**

a．普通卷烟 regular cig

b. 细支烟 slim cig

c. 两种都有 both

[Ask if Q9=b]

Q21 在参加这次调查以前你听说过电子烟吗？**Q27**

Before today, have you ever heard of e-cigarettes?

a. 听说过 yes

b. 没有 no

[Ask if Q9=a or Q21=a]

Q22 你的亲戚朋友当中现在有人用电子烟吗？**Q28**

How many of your relatives/friends are using e-cigarette?

a．没有 人用 none

b. 少数人用 few

c. 多数人用 most

d. 全部都用 all

e. 不清楚 dk

[Ask if Q9=a or Q21=a]

Copyright ©UCSD

Q23 在过去一个月里你的亲戚朋友当中有人谈论电子烟吗？**Q29**

In the past 30 days did anyone among your relatives/friends talk about e-cigarettes?

a. 有 yes

b. 没有 no

[Ask if Q23=a]

Q24 总的来说，他们对电子烟的态度是赞成还是反对？**Q30**

In general what is their attitude toward e-cigarettes?

a．多数反对 mostly negative

b．多数赞成 mostly positive

c．中立 neutral

[Ask if Q9=a or Q21=a]

Q25 在过去一个月里你见过或听到过有关电子烟的新闻报道吗？**Q31**

In the past 30 days have you heard/read any news about e-cigarettes?

a. 有 yes

b. 没有 no

[Ask if Q25=a]

Q26 总的来说，这些新闻报道对电子烟的态度是赞成还是反对？**Q32**

In general what is their attitude toward e-cigarettes?

a．多数反对 mostly negative

b．多数赞成 mostly positive

c．中立 neutral

[Ask if Q9=a or Q21=a]

Q27 你认为人们使用电子烟主要是为了。。。。。。？ **Q33**

Do you think people use e-cigarettes mainly for entertainment or quitting smoking?

a．休闲 （享受时光） entertainment

b 少吸普通卷烟 cut down regular cig.

c．戒掉普通卷烟 quit smoking regular cig.

d. 不知道

[Ask if Q9=a or Q21=a]

Q28 你向别人推荐过电子烟吗？**Q34**

Have you recommended e-cig to others?

a 推荐过 yes

b 没有 no

c. 不记得了 don’t remember

[Ask if Q28=a]

Q29 是推荐给当时在吸烟的人，已经戒烟的人，还是从未吸烟的人？（多选项，可选超过一项）**Q35**

To whom did you recommend to? (check all that apply)

a．当时在吸烟的人 current smoker

b．已经戒烟的人 former smoker

c．从未吸烟的人 never smoker

[Ask if Q9=a or Q21=a]

Copyright ©UCSD

Q30 你送过电子烟给别人吗？ Have you given e-cig to others as gift? **Q36**

a. 有 yes
b . 没有 no
c. 不记得了 don't remember

[Ask if Q30=a]

Q31 你最近一次送电子烟给别人是特别为了帮助对方戒烟还是作为普通礼物？ **Q37**

The last time you gave e-cig to others is specifically to help quit smoking or as a general gift?

a. 帮助戒烟
b. 作为普通礼物

[Ask if Q31=b]

Q32 是个人礼物还是商务礼品？ **Q38**

Was that a personal gift or business one?

a. 个人礼物
b. 商务礼品

[Everyone]

Q33 你送过香烟给别人吗？ **Q39**

Have you ever bought cigarettes for others as gifts?

a. 有 Yes
b. 没有 No
c. 不记得了 don't remember

[Ask if Q33=a]

Q34 你最近一次送的香烟是哪一种？ **Q40**

The last time you bought cigarettes for others, did you buy regular cigarettes, slim, or both?

a. 普通卷烟
b. 细支烟
c. 两种都有

[Ask if Q33=a]

Q35 是作为个人礼物还是商务礼品？ **Q41**

Was that a personal gift or business one?

a. 个人礼物
b. 商务礼品

[Ask if Q20=a or c]

Q36 过去 12 个月里你用过普通卷烟吗？ **Q42**

In the past 12 months have you used the following products?

Regular cigarettes

a. 用过 Yes
b. 没有 No

[Ask if Q20=b or c]

Q37 过去 12 个月里你用过细支烟吗？ **Q43**

In the past 12 months have you used the following products?

Copyright ©UCSD

Slim cigarettes
a. 用过 Yes
b. 没有 No

[Ask if Q9=a]

Q38 过去 12 个月里你用过电子烟吗？ 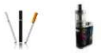 Q44
In the past 12 months have you used the following products?

E-cigs
a. 用过 Yes
b. 没有 No

[Ask if Q12=a]
Q39 过去 12 个月里你喝过酒（如啤酒，红酒，白酒）吗？ Q45
In the past 12 months have you used the following products?
Alcohol (such as beer, red wine, or white wine)
a. 用过 Yes
b. 没有 No

[Ask if Q36=a]
Q40 过去 30 天里你吸过普通卷烟吗？ Q46
In the past 30 days have you used the following products?

Regular cig
a. 用过 Yes
b. 没有 No

[Ask if Q37=a]
Q41 过去 30 天里你吸过细支烟吗？ Q47
In the past 30 days have you used the following products?
slim
a. 用过 Yes
b. 没有 No

[Ask if Q38=a]

Q42 过去 30 天里你用过电子烟吗？ 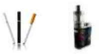 Q48
In the past 30 days have you used the following products?

E-cig
a. 用过 Yes
b. 没有 No

[Ask if Q42=a]

Q43 你用的电子烟是什么牌子？ **Q49**

What is the brand of e-cig you are using?

a. _________________(请填入电子烟品牌)

b. 不记得了

[Ask if Q39=a]

Q44 过去 30 天里你喝过酒（如啤酒，红酒，白酒）吗？ **Q50**

In the past 30 days have you used the following products?

Alcohol

a. 用过 Yes

b. 没有 No

[Ask if "Yes" to ANY of Q40, Q41, Q42, Q44, show relevant Q45-Q48] [仅显示 Q40, Q41, Q42, Q44 中答"用过"的]

过去 30 天里你有几天用过下列产品？ in the past 30 days on how many days have you used the following products?

[drop down 1-30 for each option that shows up below]

Q45 普通卷烟 regular cig **Q51** **[Show this option if YES to Q40]**

Q46 细支烟 slim **Q52** **[Show this option if YES to Q41]**

Q47 电子烟 e-cig **Q53** **[Show this option if YES to Q42]**

Q48 酒类 alcohol **Q54** **[Show this option if YES to Q44]**

（饮用酒，如啤酒，红酒，白酒。）

[Ask if Q20=c or (Q9=a & Q20=a or b or c]

Q49. 看来你吸过不止一种烟，最先开始吸的是哪一种烟? **Q55**

Which one did you start smoking first? ***coding should be consistent for Q49, & Q50***

a. 普通卷烟 regular cig ***1=普通卷烟*** ***[show if Q20=a or c]***

b. 细支烟 slim cig ***2=细支烟*** ***[show if Q20=b or c]***

c. 电子烟 e-cig, regular ***3=电子烟*** ***[show if Q9=a]***

[Ask if (Q9=a & Q20=c), show only those not selected previously]

Q50. 最后开始吸的是哪一种烟? **Q56**

Which one did you start smoking last?

a. 普通卷烟 regular cig ***1=普通卷烟*** ***[show if (Q49=b or c)]***

b. 细支烟 slim cig ***2=细支烟*** ***[show if (Q49=a or c)]***

c. 电子烟 e-cig, regular ***3=电子烟*** ***[show if (Q49=a or b)]***

[Ask if (Q20=b or c)]

Q51 你吸细支烟的主要原因是什么？ What is the main reason for you to smoke slim cig? **Q57**

a. 比普通卷烟危害小 less harmful than regular cig

b. 比普通卷烟便宜 cheaper than regular cig

c. 帮助戒掉普通卷烟 help quit regular cig

d. 少吸普通卷烟 reduce the consumption of regular cig

e. 看上去很时髦 （外观吸引人） it looks good

f. 味道不错 it tastes good

g. 其他_______________**[TEXT BOX]** other

Copyright ©UCSD

[Everyone]

Q52 **过去 30 天里**有人向你递香烟吗？ in the past 30 days did anyone invited you to smoke? **Q58**

a. 有 Yes
b. 没有 No
c. 不记得了 don't remember

[Everyone]

Q53 **过去 30 天里**有人向你递电子烟吗？ in the past 30 days did anyone invited you to use e-cig? **Q59**

a. 有 Yes
b. 没有 No
c. 不记得了 don't remember

Cigarette

[Ask if Q8=a]

Q54 到目前为止你有没有吸过至少 100 支香烟？（包括普通卷烟和细支烟） **Q60**

Have you smoked at least 100 cigarettes (including regular and slim) in your life?

a. 有 yes
b. 没有 no

[Ask if Q8=a]

Q55 你现在是每天吸，偶尔吸，还是完全不吸香烟？（包括普通卷烟和细支烟） **Q61**

Do you currently smoke every day, somedays, or not at all (including regular and slim)?

a. 每天吸 everyday
b. 偶尔吸（不是每天吸） somedays
c. 完全不吸 not at all

[Ask if Q8=a]

Q56 一年以前这个时候你吸香烟吗？（包括普通卷烟和细支烟） **Q66**

12 months ago this time did you smoke every day, somedays, or not at all (including regular and slim)?

a. 每天吸 every day
b. 偶尔吸 (不是每天吸) somedays
c.完全不吸 not at all

CIGARETTES - QUIT SECTION

[Ask if Q54=a & Q55=c & (Q36=a OR Q37=a)]

Q57 吸最后一支香烟是什么时候？ When did you smoke your last cigarettes? **Q67**

a. 过去一周内 within past 7 days
b. 过去 8-14 天 within past 8-14 days
c. 过去 15-30 天 within past 15-30 days
d. 过去 1-3 个月 within past 1-3 months
e. 过去 4-6 个月 within past 4-6 months
f. 过去 7-12 个月 within past 7-12 months

[Ask if Q54=a & Q55=c & (Q36^=a & Q37^=a)]

Q58 吸最后一支香烟是什么时候？ When did you smoke your last cigarettes? **Q68**

a. 1-2 年前

Copyright ©UCSD

b. 2-3 年前
c. 3-4 年前
d. 4-5 年前
e. 5-6 年前
f. 6-7 年前
g. 7-8 年前
h. 8-9 年前
i. 9-10 年前
j. 超过 10 年

[Ask if Q54=a & Q55=c]
Q59 你以前经常吸薄荷香烟（也称凉烟）吗？ **Q60**
Did you usually smoke Menthol cig regularly?
a. 常吸 yes
b. 不常吸 no

[Ask if Q54=a & Q55=c]
Q60 你最近一次戒烟是干戒吗（完全靠个人意志力戒烟，不用任何戒烟药物，电子烟，或其他戒烟产品）？ **Q70**
When you quit smoking the last time, did you go cold turkey (without using any quitting aids or e-cigarettes)?
a. 是 Yes
b. 不是 No

[Ask if Q60=b]
Q61 你最近一次戒烟用过戒烟药物，电子烟，或是其他戒烟产品吗？ **Q71**
Did you use medication, e-cigarettes, or other products in your last quit attempt?
a. 用过 Yes
b. 没有 no

[Ask if Q61=a]
Q62 你用了哪一种？（多选） **Q72**
Which did you use in your last quit attempt? (select all that apply)
a. 戒烟药物 （包括中药和西药） medication
b. 电子烟 e-cig ***[show option only if Q9=a]***
c. 其他 __________ （填入）

[Ask if Q62(a)=Yes]
Q63 你用了哪类药物？（多选） **Q73**
What kind of medications did you use?

a. 中药 herbal	☐ 用了 (1)	☐ 没有 (2)
b. 西药 western	☐ 用了 (1)	☐ 没有 (2)

c. 其他 ____________other
d. 不知道 dk

[Ask if Q63(b)=Yes]
Q64 你用的西药有哪些？（如使用种类超过一种请选择所有适合的选项） **Q74**
What kind of western medications did you use?

Copyright ©UCSD

a. 戒烟贴片/尼古丁口香糖 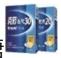 NRT

b. 悦亭 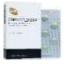 （需医生处方） zyban

c. 畅沛 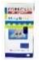 （需医生处方） Chantix

d. 其他 _________ other

e. 不记得了 can't remember

[Ask if Q64(a)=Yes]

Q65 戒烟贴片/尼古丁口香糖一共用了多久？How long have you used NRT? **Q75**

a. 不超过 7 天 7days or less

b. 超过 7 天但不到 30 天 more than 7 days but less than 30 days

c. 30 天或以上 30 days or more

d. 不记得了 dk

[Ask if Q64(b)=Yes]

Q66 悦亭一共用了多久？How long have you used Zyban? **Q76**

a. 不超过 7 天 7days or less

b. 超过 7 天但不到 30 天 more than 7 days but less than 30 days

c. 30 天或以上 30 days or more

d. 不记得了 dk

[Ask if Q64(c)=Yes]

Q67 畅沛一共用了多久？How long have you used Chantix? **Q77**

a. 不超过 7 天 7days or less

b. 超过 7 天但不到 30 天 more than 7 days but less than 30 days

c. 30 天或以上 30 days or more

d. 不记得了 dk

[Ask if Q62(b)=Yes]

Q68 电子烟一共用了多久？How long have you used e-cig that time? **Q78**

a. 不超过 7 天 7days or less

b. 超过 7 天但不到 30 天 more than 7 days but less than 30 days

c. 30 天或以上 30 days or more

d. 不记得了 dk

CIGARETTES - CURRENT USE

[Ask if Q54=a & (Q55=a OR Q55=b) & Q20=c]

Q69. 你现在吸的是普通卷烟，细支烟，还是两种都吸？ **Q79**

what kind of cigarettes do you currently smoke?

a. 普通卷烟 regular cig

b.细支烟 slim cig.

c. 两种都吸 both

Copyright ©UCSD

[Ask if (Q54=a & (Q55=a OR Q55=b) & Q20=a or b) or (Q69=a OR Q69=b)]

70 你吸香烟那天, 一天大约吸几支? Q80

___________支

[Ask if Q69=c]

Q71 你吸香烟那天, 一天大约吸几支? Q82

On average, how many cigarettes (including regular and slim) do you smoke per day?

普通卷烟: ____ 支 regular

细支烟: ____支 slim

Add to total number of cigarettes automatically: ____支 （电脑自动相加得出总支数） Q83

[Ask if Q54=a & (Q55=a OR Q55=b) & (Q69=a OR Q69=b OR Q20=a OR Q20=b)]

Q72 一般情况下你每月花多少钱买香烟? Q84

In a typical month, how much money do you spend on cigarettes?

a. 不花钱 never buy
b. 1- 100 元
c. 101-200 元
d. 201-300 元
e. 301-400 元
f. 401-500 元
g. 多于 500 元 More than 500

[Ask if Q69=C]

Q73 一般情况下普通香烟和细支烟加在一起你每月花多少钱买香烟? Q85

In a typical month, how much money do you spend **altogether** on cigarettes?

a. 不花钱 never buy
b. 1- 100 元
c. 101-200 元
d. 201-300 元
e. 301-400 元
f. 401-500 元
g. 多于 500 元 More than 500

[Ask if (Q72=b-g OR Q73=b-g)]

Q74 更详细一点，大约多少元? （如所填数字不在 Q72/Q73 所选择的范围内请提示重填） Q86

More specifically, how much money do you spend on cigarettes each month?

__________元

[Ask if Q54=a & (Q55=a OR Q55=b)]

Q75 你现在经常吸薄荷香烟（也称凉烟）吗? Do you usually smoke Menthol cigarettes? Q87

a. 常吸 yes
b. 不常吸 no

[Ask if Q54=a & (Q55=a OR Q55=b]

Q76. 过去一年里你试过戒烟吗? Have you tried to quit smoking in the past 12 months? Q88

a. 试过 yes
b. 没有 no

Copyright ©UCSD

[Ask if Q76=a]
Q77 过去一年里你有没有一次戒烟至少 24 小时？ During the last 12 months, have you quit for at least 24 hours? Q89
a．有 yes
b．没有 no

[Ask if Q77=a]
Q78 最近达到 24 小时那次你戒了多长时间？ How long did you quit the last time? Q90
a． 1-7 天 1-7 days
b．8-14 天
c．15-30 天
d．1-3 个月 1-3 months
e．4-6 个月
f．7-12 个月

[Ask if Q77=a]
Q79 你最近一次戒烟是干戒吗？（完全靠个人意志力戒烟，不用任何戒烟药物，电子烟，或其他戒烟产品） Q91
When you quit smoking the last time, did you go cold turkey (without using any quitting aids or e-cigarettes)?
1. 是 Yes
2. 不是 No

[Ask if Q79=b]
Q80 你最近一次戒烟用过戒烟药物，电子烟，或是其他戒烟产品吗？ Q92
Did you use medication, e-cigarettes, or other products in your last quit attempt?
a. 用过 Yes
b. 没有 no

[Ask if Q80=a]
Q81 你用了 哪一种？（多选） Q93
Did you use medication, e-cigarettes, or other quitting aids? (select all that apply)
a. 戒烟药物 （包括中药和西药） medication
b. 电子烟 e-cig *[show option only if Q9=a]*
c. 其他 ___________ （填入）

[Ask if Q81(a)=yes]
Q82 你用了哪类药物？(如使用种类超过一种请选择所有适合的选项) Q94
What kind of medications did you use?

a. 中药 herbal	☐ 用了 (1)	☐ 没有 (2)
b. 西药 western	☐ 用了 (1)	☐ 没有 (2)

c. 其他 _____________other
d. 不知道 dk

[Ask if Q82(b)=yes]
Q83 你用的西药有哪些？(如使用种类超过一种请选择所有适合的选项) Q95
What kind of western medications did you use?

a. 戒烟贴片/尼古丁口香糖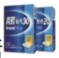 NRT

Copyright ©UCSD

b. 悦亭 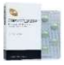 （需医生处方） zyban

c. 畅沛 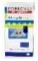 （需医生处方） Chantix

d. 其他 _________ other

e. 不记得了 can’t remember

[Ask if Q83(a)=yes]

Q84. 戒烟贴片/尼古丁口香糖一共用了多久？ How long have you used NRT? **Q96**

a. 不超过 7 天 7days or less

b. 超过 7 天但不到 30 天 more than 7 days but less than 30 days

c. 30 天或以上 30 days or more

d. 不记得了 dk

[Ask if Q83(b)=yes]

Q85 悦亭一共用了多久？ How long have you used Zyban? **Q97**

a. 不超过 7 天 7days or less

b. 超过 7 天但不到 30 天 more than 7 days but less than 30 days

c. 30 天或以上 30 days or more

d. 不记得了 dk

[Ask if Q83(c)=yes]

Q86. 畅沛一共用了多久？ How long have you used Chantix? **Q98**

a. 不超过 7 天 7days or less

b. 超过 7 天但不到 30 天 more than 7 days but less than 30 days

c. 30 天或以上 30 days or more

d. 不记得了 dk

[Ask if Q81(b)=yes]

Q87 电子烟一共用了多久？ How long have you used e-cig that time? **Q99**

a. 不超过 7 天 7days or less

b. 超过 7 天但不到 30 天 more than 7 days but less than 30 days

c. 30 天或以上 30 days or more

d. 不记得了 dk

[Ask if Q54=a & (Q55=a OR Q55=b]

Q88 你打算戒掉香烟吗？ **Q100**

Do you plan to quit smoking cigarettes?

a. 有打算，一个月内 yes, within one month

b. 有打算，6个月内 yes, within 6 months

c. 有打算，6个月以后 yes, after 6 months

d. 没有打算 no

[Ask if Q54=a & (Q55=a OR Q55=b]

Q89 如果将来你打算戒烟，你会用戒烟药物（如戒烟贴片，畅沛，悦亭）来帮助戒烟吗？ **Q101**

If you are going to quit smoking in the future, will you use quit medicine (such as nrt, Chantix, or zyban) to help you quit?

a. 会 yes

b. 不会 no
c. 不知道 dk

[Ask if Q54=a & (Q55=a OR Q55=b]

Q90 你的亲友对你抽香烟怎么看？ **Q102**

How do your family and friends feel about your use of cigarettes?

a．多数反对 mostly negative
b．多数赞成 mostly positive
c．中立 neutral

电子烟

Never

[Ask if Q9=b]

Q91 如果朋友请你试用电子烟，你会接受吗？If your friend invites you to try e-cig will you take it? **Q103**

a. 肯定会 definitely yes
b. 可能会 maybe
c. 可能不会 maybe not
d. 肯定不会 definitely not

Ever ecig

[Ask if Q9=a]

Q92 用电子烟有没有用过至少 10 天（不管有没有连续使用）？ Have you used e-cig on at least 10 days? **Q104**

a．有 yes
b．没有 no

[Ask if Q92=a]

Q93 你有过至少一个月天天使用电子烟吗？ **Q105**

Have you ever used e-cig daily for at least 1 month?

a．有 yes
b．没有 no
c. 不记得了

[Ask if Q9=a]

Q94 你第一次用的电子烟是从哪里得到的？ The first time you used an e-cigarette, where did you get it? **Q106**

a．自己买的 bought yourself
b．厂商赠品或品吸 sample from manufacturer
c．亲友给的 from relatives/friends
d．在其他人的电子烟抽了一口 （好奇） puffed from other's e-cig
e．其他 _______ **[TEXT BOX]** Other
f. 不记得了 dk

[Ask if Q9=a]

Q95 你用电子烟的主要原因是什么？What was your main reason for using e-cig? **Q107**

a．味道不错 it tastes good

Copyright ©UCSD

b．比香烟危害小 less harmful than cig
c．比香烟便宜 cheaper than cig.
d．用来帮助戒烟 to help quit smoking cig.
e．可以在禁烟的场合使用 used in places where smoking is prohibited
f．看上去不错 looks good
g. 好奇 curiosity
h．和亲友聚会时用 hang out with friends
i．控制体重 weight control
j．其他 ________________**[TEXT BOX] [Prompt]** other

[Ask if Q9=a]
Q96 你现在用电子烟是每天用，偶尔用，还是完全不用？ **Q108**
Do you currently use e-cig every day, somedays, or not at all?
a．每天用 every day
b．偶尔用(不是每天用) somedays
c．完全不用 not at all

[Ask if Q9=a]
Q97 一年前这个时候你用电子烟吗？ **Q110**
12 months ago did you use e-cig every day, somedays, or not at all?
a．每天用 every day
b．偶尔用(不是每天用) somedays
c．完全不用 not at all

[Ask if Q96=c & Q38=a]
Q98 你最后一次用电子烟是什么时候？ **Q111**
How long ago did you last use e-cig?
a．过去一周内 within past 7 days
b．过去 8-14 天内 8-14 days
c．过去 15-30 天内 15-30 days
d．过去 1-3 个月内 1-3 months
e．过去 4-6 个月内 3-6 months
f．过去 7-12 个月内 6-12 months

[Ask if Q96=c & Q38=b]
Q99 你最后一次用电子烟是什么时候 **Q112**
How long ago did you last use e-cig?
a. 过去 1-2 年内 1-2 years
b. 过去 2-3 年内
c. 过去 3-4 年内
d. 过去 4-5 年内
e.过去 5-6 年内
f. 过去 6-7 年内
g. 过去 7-8 年内
h. 过去 8-9 年内
i. 过去 9-10 年内
j. 超过 10 年 more than 10 years

[Ask if Q96=c]

Q100 让你停止使用电子烟的主要原因是什么？ **Q113**

What is the main reason you stopped using e-cig?

a. 味道不好 — it tastes not good
b. 价钱太贵 — expensive
c. 对戒烟没帮助 — did not help quit smoking cig
d. 不想上瘾 — don't want to be addicted
e. 亲友不喜欢 — relatives/friends don't like it
f. 含有害物质，不利健康 — contains harmful ingredients, not healthy
g. 最近刚刚开始抽 — just started using recently
h. 对产品质量不满意 — not satisfied with the quality of the products
i. 使用不方便 (比如加烟油很麻烦，吸烟时很费力) — inconvenient to use (difficult to refill, hard to puff etc.)
j. 公共场合不允许使用 — not allowed in public places
k. 其他 ________**[TEXT BOX]** — other

[Ask if Q96=a or b]

Q101 每个人使用电子烟的方式不同。有人一天之内从早到晚不间断的用，有人像吸烟那样隔一段时间用一次。你主要是怎么用的？ **Q114**

People use e-cig in different style: some people use e-cig all day long, others use them with breaks in between. Which way do you mainly use them?

a．一天之内从早到晚不间断的用 — all day long
b．隔段时间用一次 — with breaks in between

[Ask if Q96=a]

Q102 通常每天要用多少次？(每次哪怕吸一口) **Q115**

(Daily) How many separate times do you usually use them each day (even one puff or hit)?

a．不超过 20 次 — 20 times or less
b．超过 20 次 — more than 20 times

[Ask if Q96=b]

Q103 在你用电子烟的时候, 通常一天要用多少次？(每次哪怕吸一口) **Q116**

(Nondaily) On the days you use e-cig, how many separate times do you usually use them (even one puff or hit)?

a．不超过 20 次 — 20 times or less
b．超过 20 次 — more than 20 times

[Ask if Q102=a OR Q103=a]

Q104 更详细一点，你大约用几次？more specifically, how many separate times do you use them? **Q117**

a．1 次
b．2-3 次
c．4-5 次
d．6-10 次
e．11-15 次
f．16-20 次

[Ask if Q102=b OR Q103=b]

Q105 更详细一点，你大约用几次？more specifically, how many separate times do you use them? **Q118**

a．21-25 次
b. 26-30 次

c．31-35 次
d．36-40 次
e．超过 40 次　　more than 40

[Ask if Q96=a or b]
Q106 每次用电子烟时你大约会吸几口？ Approximately how many puffs (hits) do you take each time? **Q119**
a．1 口
b．2-3 口
c．4-5 口
d．6-10 口
e．11-15 口
f．16-20 口
g．21-25 口
h．26-30 口
i．超过 30 口　　more than 30 puffs

[Ask if Q96=a or b]
Q107 你现在常用的电子烟的烟油中含有尼古丁吗？ Does the e-cig juice you use most often contains nicotine? **Q120**
a. 有　　yes
b. 没有　　no
c. 不知道　　dk

[Ask if Q107=a]
Q108 电子烟油中尼古丁的含量是多少？ What strength e-cig do you use most often? **Q121**
a．低浓度，（10 毫克或更低）　　light: 10mg or less
b．中等浓度，（11-17 毫克）　　medium: 11-17mg
c．高浓度，（18-23 毫克）　　strong: 18-23mg
d．极高浓度，（24 毫克或更高）　　extra strong: 24mg or more
e．不知道　　dk

[Ask if Q96=a or b]
Q109 你最初开始用电子烟的时候烟油中含有尼古丁吗？ **Q122**
When you started using e-cigarettes, did the e-cig juice you used most often contain nicotine?
a. 有　　yes
b. 没有　　no
c. 不知道　　dk

[Ask if Q109=a]
Q110 那时用的电子烟油中尼古丁的含量是多少？ **Q123**
When you started using e-cigarettes, what strength were you using most often?
a．低浓度，（10 毫克或更低）　　light: 10mg or less
b．中等浓度，（11-17 毫克）　　medium: 11-17mg
c．高浓度，（18-23 毫克）　　strong: 18-23mg
d．极高浓度，（24 毫克或更高）　　extra strong: 24mg or more
e．不知道　　dk

[Ask if Q96=a or b]

Copyright ©UCSD

Q111 你现在最喜欢哪一种口味的电子烟？What is your favorite e-cig flavor? **Q124**

a．酒味 alcohol
b．咖啡味 coffee
c．糖果/点心味（如香草，巧克力） dessert/candy
d．水果味 fruit
e．薄荷味 menthol
f．烟草味 tobacco
g．烟草薄荷混合味 tobacco-menthol mix
h．其他 ____________**[TEXT BOX] [Prompt]** other
i．无所谓 no favorite

[Ask if Q96=a or b]
前面你曾经提到过现在使用电子烟。电子烟使用烟油，还可以进一步分为封闭型和开放型。
Regular e-cigs basically can be classified as closed and open systems

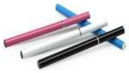

- 封闭型的**特点是不能自己加注烟油**。(有些为一次性使用，有些可以换烟弹)
  Closed system: disposable or pre-filled cartridge system. You do not add your own e-liquid

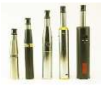

- 开放型的共同**特征是可以自己加注烟油**，反复使用。
  Any system you refill using bottles of e-liquid.

[Ask if Q96=a or b]
Q112 你现在最常用的是封闭型还是开放型的？ **Q125**
You said that you have used “steam” e-cigarettes. Are you currently using a closed-system or open-system MOST OFTEN?

a. 封闭型 closed
b. 开放型 open
c. 一半一半 Use both closed and open systems equally

[Ask if Q96=a or b]
Q113 最早开始用电子烟时用的是封闭型还是开放型的？ **Q126**
When you started using regular e-cigarettes, was that a closed-system or open-system?

a. 封闭型 closed
b. 开放型 open

CLOSED-SYSTEM

[Ask if Q112=a or c]
Q114 你现在使用封闭型电子烟每周超过一支吗？ **Q127**
You said you currently use closed system e-cigarettes. Do you usually use more than 1 closed system e-cig each week?

a．超过 yes
b．没有 no

Copyright ©UCSD

[Ask if Q114=a]

[Number box; Range 1-30]

Q115. 具体来说，每周要用几支？ __________ Q128

How many do you usually use each week?

[Ask if Q114=b]

[Number box; Range 1-9]

Q116 每个月要用几支？ __________ How many do you usually use each month? Q129

OPEN-SYSTEM

[Ask if Q112=b or c]

Q117 你现在使用开放型电子烟（可以手工加注烟油），每周需要加注烟油一次以上吗？ Q130

You said you currently use open system e-cigarettes. Do you usually refill your e-cig tank more than 1 time each week?

a．需要 yes

b．没有 no

[Ask if Q117=a]

Q118 你每周要用多少毫升电子烟油？About how much e-liquid do you use each week? Q131

a．不超过 3 毫升 3ml or less

b．4-5 毫升

c．6-10 毫升

d．11-15 毫升

e．16-20 毫升

f．21-25 毫升

g．26-30 毫升

h．31-35 毫升

i．36-40 毫升

j．超过 40 毫升 more than 40ml

k．不知道 dk

[Ask if Q117=b]

Q119 你每月要用多少毫升电子烟油？About how much e-liquid do you use each month? Q132

a．不超过 3 毫升 3ml or less

b．4-5 毫升

c．6-10 毫升

d．11-15 毫升

e．16-20 毫升

f．21-25 毫升

g．26-30 毫升

h．31-35 毫升

i．36-40 毫升

j．41-50 毫升

k．超过 50 毫升 more than 50ml

l．不知道 dk

[Ask if Q96=a or b]

Q120 你用的电子烟或烟油一般是从哪里得到的？Where do you usually get your e-cigs or e-liquid? Q133

Copyright ©UCSD

a．网购 internet
b．药房 Pharmacy
c．加油站/便利店 gas station/convenient store
d．超市 grocery store/supermarket
e．商场内出售电子烟的柜台 shopping mall kiosk
f．烟草店 smoke shop
g．电子烟专卖（实体）店 e-cig/vape shop
h．亲友给的 relatives/friends
i. 国外旅行带回 From a overseas trip
j．从未买过电子烟 never bought
k．其他 __________**[TEXT BOX] [Prompt]** other

[Ask if Q112=b or c]
Q121 你开始用开放型电子烟的时候花了多少钱购买所需要的设备？（如亲友赠送，请填 0） **Q134**
___________元

[Ask if Q112=b or c]
Q122 不包括最初购买设备的花销，你现在每个月大约花多少钱买电子烟烟油和附件？ **Q135**
Excluding startup costs, in a typical month, how much money do you spend on e-cig products (including all)?
a. 不花钱 never bought
b. 1- 100 元
c. 101-200 元
d. 201-300 元
e. 301-400 元
f. 401-500 元
g. 多于 500 元 More than 500

[Ask if Q112=a]
Q123 你现在每个月大约花多少钱买电子烟？ **Q136**
In a typical month, how much money do you spend on e-cig products (including all)?
a. 不花钱 never bought
b. 1- 100 元
c. 101-200 元
d. 201-300 元
e. 301-400 元
f. 401-500 元
g. 多于 500 元 More than 500

[Ask if Q122=b-g OR Q123=b-g]
Q124 更详细一点，大约多少元？（如所填数字不在 Q122/Q123 所选择的范围内请提示重填） **Q137**
More specifically, how much do you spend on e-cig products each month?
__________元

[Ask if Q96=a or b]
Q125 有没有试过戒掉（或停用）电子烟？ Have you ever tried to stop using e-cig? **Q138**
a．有 yes
b．没有 no

Copyright ©UCSD

[Ask if Q125=a]

Q126 过去一年里有没有试过戒掉 (停用) 电子烟? Q139

Have you ever tried to stop using e-cig in the past 12 months?

a．有 yes

b．没有 no

[Ask if Q126=a]

Q127 你想要戒掉（停用）电子烟的主要原因是什么？Q140

What was the main reason you tried to stop using e-cig?

a. 味道不好 tastes not good

b. 价钱太贵 expensive

c. 对戒烟没帮助 did not help quit smoking cig

d. 不想上瘾 don't want to be addicted

e. 亲友不喜欢 relatives/friends did not like it

f. 含有害物质，不利健康 contains harmful ingredient, not healthy

g. 最近刚刚开始抽 just started using

h. 对产品质量不满意 not satisfied with the quality of the product

i. 使用不方便 (比如加烟油很麻烦，吸烟时很费力) inconvenient to use (difficult in refill, hard to puff etc.)

j. 公共场合不允许使用 not allowed in public places

k. 其他 ________**[TEXT BOX]** other

[Ask if Q96=a or b]

Q128 你有打算戒掉（停用）电子烟吗？Q141

Do you plan to stop using e-cig?

a．有打算， 一个月内。 Yes, within one month

b．有打算，六个月内 Yes, within six month

c．有打算，6个月以后 Yes, after six month

d．没有打算 no

[Ask if Q128=a-c]

Q129 你打算戒掉（停用）电子烟的主要原因是什么？What is the main reason you want to stop using e-cig? Q142

a. 味道不好 tastes not good

b. 价钱太贵 expensive

c. 对戒烟没帮助 do not help quit smoking cig

d. 不想上瘾 don't want to be addicted

e. 亲友不喜欢 relatives/friends do not like it

f. 含有害物质，不利健康 contains harmful ingredient, not healthy

g. 最近刚刚开始抽 just started using

h. 对产品质量不满意 not satisfied with the quality of the product

i. 使用不方便 (比如加烟油很麻烦，吸烟时很费力) inconvenient to use (difficult in refill, hard to puff etc.)

j. 公共场合不允许使用 not allowed in public places

k. 其他 ________**[TEXT BOX]** other

[Ask if Q96=a or b]

Q130 你的亲友对你用电子烟怎么看？How do your family members/friends feel about your use of e-cig? Q143

a．多数反对 mostly negative

Copyright ©UCSD

b．多数赞成　　mostly positive
c．中立　　neutral

[Ask if (Q96=a or b) & (Q55=a or b) & Q54=a]

Q131 自从使用电子烟后你对戒掉香烟的兴趣。。。 **Q144**

Since started using e-cig, your interests in quitting regular cigarettes has..

a. 增加了　　increased
b. 减少了　　decreased
c. 没有变化　stayed the same

[Ask if (Q96=a or b) & (Q55=a or b) & Q54=a]

Q132 自从使用电子烟后你吸烟的支数。。。 **Q145**

Since started using e-cig, the number of regular cigarettes you smoke has..

a. 增加了　　increased
b. 减少了　　decreased
c. 没有变化　stayed the same

Q133 你现在住在哪个城市？ **Q146**

Which city are you living now?

[If Q10=1 or Q11=1: end of the formal survey]　（凡用过无烟烟草[Q10=1]或尼古丁饮料[Q11=1]：不再回答Q133-Q150 但请继续问Q150以后E-Panel增加的问题如年龄，收入，教育，职业等。If ever used SLT or nicotine drink, end of the formal survey but continue on E-Panel added questions: age, income, education, career, etc.）

[Ask if Q10=2 and Q11=2] ***（没用过无烟烟草，也没用过尼古丁饮料的：继续回答以下问题. Only those who never used SLT and nicotine drink will continue）***

[Ask if Q10=2 and Q11=2]

Q134 你的性别是。。 **Q147**

What is your gender?

Randomization is stratified by city and gender….

[Ask if Q10=2 and Q11=2]

Q135 ***随机显示以下 a,b,c 的信息内容，比例 1:1:1(以答题者所在城市及性别为模块进行随机分组，比如所有来自北京的应答者分为男，女两个模块，在此基础上随机分组)。*** **Q148**

[randomly assign the respondents into either a or b or c group stratified by the city where respondent lives and gender]

a. 关于电子烟的研究在学术界很有争议。西方国家权威卫生机构（如英国皇家医学会和美国疾病控制中心）最近相继发表报告。其中一篇总结报告指出：1. 电子烟所含的有害物质比普通香烟少很多(只有香烟的 5%)。 2. 电子烟可以帮助吸烟者戒烟。**(标注 Q135= a)**

E-cigarettes are a controversial topic, and researchers have different views. Recently some highly respected health authorities in western countries (such as British Royal Medical Association, U.S. CDC) published their latest reports about e-cigarette research. One of them pointed out: 1. E-cig contains much less harmful ingredient than regular cigarette does (less than 5% of regular cig). 2. E-cigarettes can help smokers quit. (old)

E-cigarettes are a controversial topic, and researchers have different views. Recently some highly respected health authorities in western countries (such as the British Royal Medical Association and the U.S. CDC) published their latest report about e-cigarette research. One of the summaries stated: 1. E-cigarettes contain a lot less of harmful substances compared to regular cigarettes (only 5% that of cigarettes). 2. E-cigarettes can help smokers quit. (Edited)

Copyright ©UCSD

b. 关于电子烟的研究在学术界很有争议。西方国家权威卫生机构（如英国皇家医学会和美国疾病控制中心）最近相继发表报告。其中一篇总结报告指出：1. 电子烟中含有多种有害物质(包括尼古丁，二乙酰，超微颗粒，苯，镍，锡，铅等)。2. 虽然有人主张用电子烟来帮助吸烟者戒烟，但没有足够的科学数据证明电子烟有效。国家食品和药物管理机构也没有批准使用电子烟作为戒烟工具。**(标注 Q135= b)**

E-cigarettes are a controversial topic, and researchers have different views. Recently some highly respected health authorities in western countries (such as British Royal Medical Association, U.S. CDC) published their latest reports about e-cigarette research. One of them pointed out: 1. E-cigs contain many harmful substances. 2. Although some recommend it as an aid to quit smoking, there is insufficient scientific data to support its effectiveness. In addition, E-cigarettes are not approved by the FDA as a smoking cessation aid. (old)

E-cigarettes are a controversial topic, and researchers have different views. Recently some highly respected health authorities in western countries (such as the British Royal Medical Association and the U.S. CDC) published their latest report about e-cigarette research. One of the summaries stated: 1. E-cigarettes contain many harmful substances (including nicotine, diacetyl, ultrafine particles, benzene, tin, lead, etc). 2. Although some people recommend e-cigarettes as an aid to quit smoking, there is insufficient scientific data to support its effectiveness. The National Food and Drug Administration has not approved to use of e-cigarettes as a smoking cessation aid. (Edited) 02/18/2020 by Jessica.

c. 无信息显示，直接跳至 **Q137**。**(标注 Q135= c)** （这是对照组）

No message shown, go to Q137 directly（control group）

[Ask if Q135 = a or b]

Q136 你觉得这些研究结论的可信度有多高？How dependable do you feel these research conclusions are? **Q149**

a. 完全可信 completely dependable
b. 比较可信 mostly dependable
c. 比较不可信 more undependable
d 完全不可信 completely undependable
e. 无法判断 No way to judge (neutral)

[Ask if Q10=2 and Q11=2]

你觉得经常使用下列产品对个人的健康危害有多大？

How harmful is regular use of the following products to the health of individuals who use them?

	完全无害 Completely harmless 1	2	3	4	5	6	7	8	9	极度有害 extremely harmful 10
Q137 普通卷烟 **Q150 cig**	☐	☐	☐	☐	☐	☐	☐	☐	☐	☐
Q138 细支烟 **Q151 slim**	☐	☐	☐	☐	☐	☐	☐	☐	☐	☐
Q139 电子烟 **Q152 e-cig**	☐	☐	☐	☐	☐	☐	☐	☐	☐	☐

程序员请注意：有关 Q137 – Q139 问题的显示顺序：(Notes for programmer): abc, acb…
Q137(a,普通卷烟)总是第一个出现， abc, acb

[Ask if Q10=2 and Q11=2]

Q140 你觉得电子烟能用来帮助戒掉香烟吗？ **Q153**

Do you feel e-cigarettes can be used to help quit smoking cigarettes?

Copyright ©UCSD

a. 能 yes
b. 不能 no
c. 不知道 dk

[Ask if Q10=2 and Q11=2]
Q141 你觉得我国食品药品监督管理机构已经批准使用电子烟来帮助戒烟了吗？ Q154
Do you think the national FDA has approved the use of e-cigarettes to help quitting?
a．已批准 yes
b．没有 no
c. 不知道 dk

[Ask if Q141=b or c]
Q142 你觉得我国食品药品监督管理机构应该批准使用电子烟来帮助戒烟吗？ Q155
Do you think the national FDA should approve the use of e-cig to help quitting?
a．应该 yes
b．不应该 no

[Ask if Q10=2 and Q11=2]
Q143 你觉得未来 5 年里吸烟的人数会增加，减少，还是不变？ Q156
Over the next 5 years, do you think the number of cigarette smokers will increase, decrease, or stay the same?
a. 增加 increase
b. 减少 decrease
c. 不变 stay the same

[Ask if Q10=2 and Q11=2]
Q144 你觉得未来 5 年里使用电子烟的人数会增加，减少，还是不变？ Q157
Over the next 5 years, do you think use of e-cigarettes will increase, decrease, or stay the same?
a. 增加 increase
b. 减少 decrease
c. 不变 stay the same

[Ask if (Q10=2 & Q11=2) and (Q55=a or b) & Q54=a]
Q145 如果将来你打算戒烟，你会用电子烟来帮助戒烟吗？ Q158
If you plan to quit smoking in the future, will you use e-cigarettes to help you quit?
a. 会 yes
b. 不会 no

[Ask if Q10=2 and Q11=2]
Q146 总体来讲，您的健康状况是。。。 Q159
a. 非常好 excellent
b. 很好 very good
c. 好 good
d. 一般 average
e. 差 poor

[Ask if Q10=2 and Q11=2]

Copyright ©UCSD

最后几个问题。。
Last couple of questions....

近几年国内外厂商研发成功一种"加热不燃烧" 烟。这种烟使用特别设计的电子装置用电池加热烟丝但不燃烧。主要有两类：一类使用特制的烟弹，如

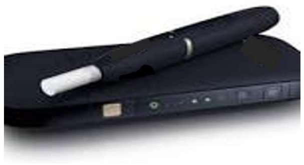

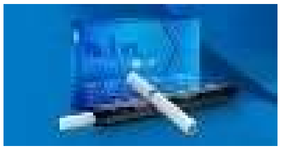

另一类使用普通卷烟（真烟），如

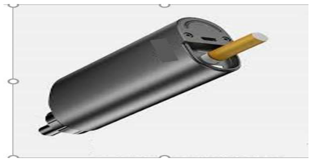

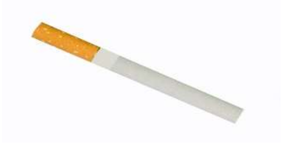

In the last few years, tobacco companies developed a new type of tobacco products called "heat not burn". There are mainly two major categories: one uses specially made heat sticks, such as IQOS. The other uses regular cigarette, such as IUOC. They all use battery to heat tobacco leaf instead of burning.

The imported products are not currently available on the market while domestic ones are sold everywhere.

[Ask if Q10=2 and Q11=2]

Q147 你用过这类 "加热不燃烧"烟 吗？ **Q160**

Have you ever used heat not burn tobacco?

a. 用过 Yes
b. 没有 No

[Ask if Q147=a]

Q148 过去30天里你用过吗？ **Q161**

Have you used it in the past 30 days?

a. 用过 Yes
b. 没有 No

[Ask if Q148=a]

Q149 你用的是什么牌子？ **Q162**

Which brand did you use?

a. _______________ （请填入品牌）
b. 不记得了 don't remember

[Everyone]

Q150 我们希望能在一年以后再次请你发表意见，了解你对上述问题的看法是否有改变。完成访问后我们会为您提供一定金额的手机充值或手机流量以表达我们的感谢。你愿意参与吗？请您留下可供充值的手机号码。

We would like to contact you after one year to find out if your opinion on these issues has changed. After completing the

Copyright ©UCSD

Follow-up survey we will recharge your cell phone with extra calling minutes or data volume to show our appreciation. Is that okay for us to contact you next year? **Q163**

a. 愿意 （请您留下可供充值的手机号码：___________________） yes
b. 不愿意 no

您已经回答了所有的问题。非常感谢您的参与！！
You have answered all the questions. Thank you for your participation!

Q165: AGE = code + 16
Q166: PERSONAL INCOME
1 no income
2 =<1000
3 1000-1999
4 2000-2999
5 3000-3999

11 9000-9999
12 10000-19999

15 >=40000
16 Refused

Q167: HOUSE INCOME
1 - 14 SAME AS Q166
15 40000 – 49999
16 50000 – 59999
17 >=60000
18 Refused

Q168: EDUCATION
1 Middle school or less
2 High school/vocational school
3 Some college
4 BA/BS
5 MS/MA+

Q169: MARITAL STATUS
1 Single
2 Has boy/girlfriend but not live together
3 Lived together but not married
4. Married
5. Divorced
6. Other

Q170: JOB CATEGORY
Q171_1 – Q171_10: Waves of Survey
